# Combined network pharmacology and virtual reverse pharmacology approaches for identification of potential targets to treat vascular dementia

**DOI:** 10.1038/s41598-019-57199-9

**Published:** 2020-01-14

**Authors:** Alexey A. Lagunin, Sergey M. Ivanov, Tatyana A. Gloriozova, Pavel V. Pogodin, Dmitry A. Filimonov, Sandeep Kumar, Rajesh K. Goel

**Affiliations:** 1 Pirogov Russian National Research Medical University, Department of Bioinformatics, Moscow, 117997 Russia; 2 Institute of Biomedical Chemistry, Department of Bioinformatics, Moscow, 119121 Russia; 3 Punjabi University, Department of Pharmaceutical Sciences and Drug Research, Patiala, 147002 India

**Keywords:** Computational biology and bioinformatics, Dementia, Cheminformatics

## Abstract

Dementia is a major cause of disability and dependency among older people. If the lives of people with dementia are to be improved, research and its translation into druggable target are crucial. Ancient systems of healthcare (Ayurveda, Siddha, Unani and Sowa-Rigpa) have been used from centuries for the treatment vascular diseases and dementia. This traditional knowledge can be transformed into novel targets through robust interplay of network pharmacology (NetP) with reverse pharmacology (RevP), without ignoring cutting edge biomedical data. This work demonstrates interaction between recent and traditional data, and aimed at selection of most promising targets for guiding wet lab validations. PROTEOME, DisGeNE, DISEASES and DrugBank databases were used for selection of genes associated with pathogenesis and treatment of vascular dementia (VaD). The selection of new potential drug targets was made by methods of NetP (DIAMOnD algorithm, enrichment analysis of KEGG pathways and biological processes of Gene Ontology) and manual expert analysis. The structures of 1976 phytomolecules from the 573 Indian medicinal plants traditionally used for the treatment of dementia and vascular diseases were used for computational estimation of their interactions with new predicted VaD-related drug targets by RevP approach based on PASS (Prediction of Activity Spectra for Substances) software. We found 147 known genes associated with vascular dementia based on the analysis of the databases with gene-disease associations. Six hundred novel targets were selected by NetP methods based on 147 gene associations. The analysis of the predicted interactions between 1976 phytomolecules and 600 NetP predicted targets leaded to the selection of 10 potential drug targets for the treatment of VaD. The translational value of these targets is discussed herewith. Twenty four drugs interacting with 10 selected targets were identified from DrugBank. These drugs have not been yet studied for the treatment of VaD and may be investigated in this field for their repositioning. The relation between inhibition of two selected targets (GSK-3, PTP1B) and the treatment of VaD was confirmed by the experimental studies on animals and reported separately in our recent publications.

## Introduction

Dementia is a group of neurological diseases that usually occur in old age and are characterized by a progressive loss of cognitive function, leading to deterioration in social and professional abilities associated with memory loss, learning, communication, and reasoning. Ten million people develop dementia every year. According to Global Dementia Observatory (GDB) the number of people with dementia will increase from 50 million to 152 million by 2050. It is expected that the annual global cost of dementia will be doubled by 2030 up to US$ 2 trillion^1^.

The most common form of dementia is Alzheimer’s disease, which accounts 60–70% of cases. Vascular dementia (VaD) is the second common form of dementia with significantly high risk of ischemic stroke^2^. Compared to Alzheimer’s disease, VaD receives relatively little research attention. Despite the suffering of patients and their families, the short life expectancy (the median survival time is approximately 3.3 years), and the social burden on society, no drug therapy has been approved for its treatment.^3^. The number of known drug targets is not enough for development of new effective medicines for the treatment of VaD^4^. Progress in this research area has been difficult as the exact nature of the relation between cerebrovascular pathology and cognitive impairment is not well understood^5^. Growing evidence suggests that vascular dementia is attributable to various risk factors including age, physical inactivity, obesity, diabetes mellitus, hypertension, high cholesterol, cigarette smoking, alcohol and tobacco use^6^.

The successful use of big data in drug discovery has been demonstrated in past few years. Therefore, it is reasonable why researchers are devoting their efforts toward building interactome, over wet lab experiments as they are time-consuming and costly. Interactome is a molecular interaction network of disease, and may provide mechanistic information of selected disease^7,8^. Although, interaction between network partners can reveal novel targets however, diseased networks did not directly give idea of best target selection, which instead was the major goal of our framework. Creation of the interactome followed by selection of precise and druggable targets is clearly work to do. It is a multidimensional process which requires interplay between system approaches that may be driven by traditional knowledge.

Ancient systems of healthcare (Ayurveda, Siddha, Unani and Sowa-Rigpa) have been used from centuries for the treatment of vascular diseases and dementia. However, the ingredients of plant extracts are complex (i.e., they may contain hundreds of compounds), and their mechanisms of action are not clearly understood^9–12^. The information is diverse, and if collected and analyzed strategically, can fish targets from the interactome through robust interplay of network pharmacology (NetP) with reverse pharmacology (RevP). A key insight in this framework is that phytomolecules with known structure and biological activities are likely to interact with most promising target and thus, may guide target selection without ignoring mainstream path of transforming big data into interactome. This approach may reduce time, money and effort of drug development, to provide novel targets for guiding wet lab experiments.

**Figure 1:**
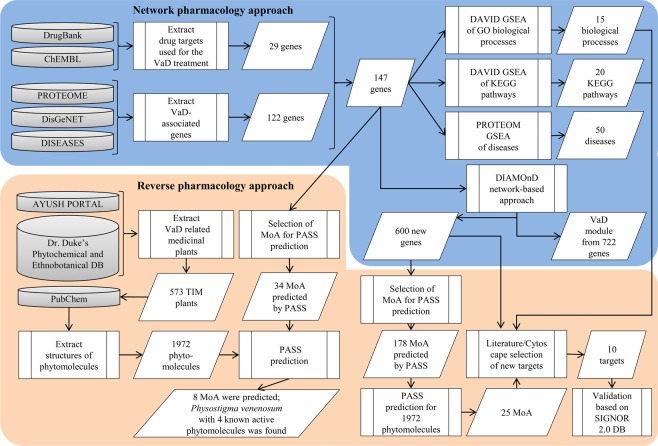
General workflow of the study. MoA – Mechanism of Action, GSEA – Gene Set Enrichment Analysis.

To this end, this work demonstrates collection, analysis and transformation of cutting edge data into the interactome using methods of NetP (enrichment analysis of Gene Ontology biological processes and KEGG pathways, DIAMOnD algorithm) followed by collection of the traditional knowledge and target fishing from the interactome through lens of RevP using PASS (Prediction of Activity Spectra for Substances) software (Fig. 1). Selected targets may guide wet lab experiments or targeted by therapies for the treatment of vascular dementia by repurposing.

## Methods

### Databases

The following databases were used to identify genes associated with VaD, TIM (Traditional Indian Medicine) plants and phytochemicals with known structures and pharmacological properties:

- PROTEOME database contains curated relationships between genes from humans or animals and diseases (https://portal.biobase-international.com/build_ghpywl/idb/1.0/html/bkldoc/index.html).
- DisGeNET (http://www.disgenet.org/web/DisGeNET/)^13^, Disease-Connect (http://disease-connect.org/)^14^ and DISEASES (http://diseases.jensenlab.org/)^15^ databases contain information about relationships between human/animal genes and diseases that was accumulated from various sources mainly through text-mining approaches.
- DrugBank (http://www.drugbank.ca/)^16^ and ChEMBL (https://www.ebi.ac.uk/chembl/)^17^ databases were used to identify drug targets associated with VaD treatment.
- AYUSH RESEARCH PORTAL includes knowledge of Ayurveda, Yoga-Naturopathy, Unani, Siddha, Homeopathy and Sowa-Rigpa (http://ayushportal.nic.in/Default.aspx).
- Dr. Duke’s Phytochemical and Ethnobotanical database includes data from plant, chemical, biological and ethnobotany searches (https://phytochem.nal.usda.gov/phytochem/help/index/about).
- PubChem (https://pubchem.ncbi.nlm.nih.gov/), which is the largest publicly available chemical database, was used for identifying the structures of phytomolecules^18^.

### Computational tools

The following computational tools and resources were used in the study:

- Progenesis SDF studio software (http://www.nonlinear.com/progenesis/sdf-studio/), which is free software to view and edit SDF and MOL files.
- KEGG pathways database (https://www.genome.jp/kegg/pathway.html) is a part of KEGG (Kyoto Encyclopedia of Genes and Genomes) is a free available resource, which particularly contains information on more than three hundreds signaling and metabolic pathways reflecting normal and disease states, e.g. “MAPK signaling pathway”, “apoptosis”, “vascular smooth muscle contraction”, and “Alzheimer disease”^19^.
- Gene Ontology (http://geneontology.org) is a knowledgebase of gene functions, which consists of three ontologies: biological function, molecular function and cellular component^20^.
- DAVID (The Database for Annotation, Visualization and Integrated Discovery), is a web service with tools for functional annotation of genes including the gene set enrichment analysis (https://david.ncifcrf.gov/)^21^.
- DIAMOnD (DIseAse MOdule Detection) is an algorithm for identification of network modules associated with diseases^22^.
- SIGNOR (SIGnaling Network Open Resource) 2.0 database is a free available resource including graphical representations of directed interactions between proteins (https://signor.uniroma2.it/)^23^.
- PASS (Prediction of Activity Spectra for Substances) software is a tool for computational prediction of biological activity spectra for substances based on their structural formulas (http://www.way2drug.com/PASSOnline/)^24^.

### Structures of phytomolecules

A total of 573 TIM plants that have been used for several centuries to treat dementia and associated risk factors were selected from the ancient Indian system of healthcare (i.e., Ayurveda, Siddha, Unani and Sowa-Rigpa), and these plants are considered to be safe and effective. We focused on specific risk factors such as diabetes, hypertension, high cholesterol, etc., because they are strongly associated with several vascular pathologies and events leading to VaD.

**Figure 2:**
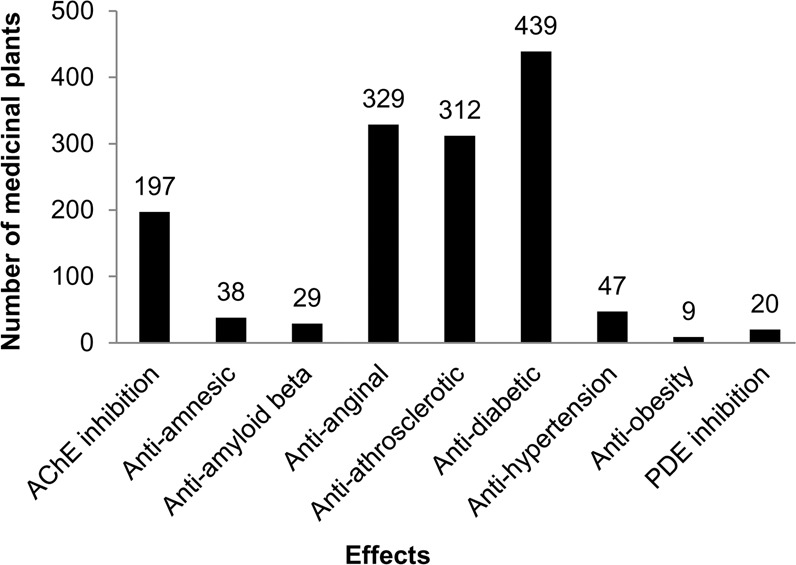
Number of medicinal plants related to dementia treatment and associated diseases. AChE – acetylcholinesterase; PDE – phosphodiesterase.

A special set of 2D-structures of 1972 main phytomolecules from the 573 medicinal plants was created as SD file using Progenesis SDF studio software. The set includes only single electroneutral organic structures with molecular weights ranging from 50 to 1250 Da and at least 3 carbon atoms to meet the requirements to use PASS (Prediction of Activity Spectra for Substances) software, which was utilized to predict the interactions of phytochemicals with selected targets (see below). A diagram with the number of medicinal plants used for the treatment of memory disorders and diseases leading to the development of dementia is shown in Fig. 2.

Some medicinal plants are simultaneously used for the treatment of different diseases. The lists of medicinal plants and phytomolecules are provided in the Supplements section (Data file S2 and S4, respectively).

### Gene set enrichment analysis

Gene set enrichment analysis is a method for identifying classes of genes or proteins that are over-represented in a large set of genes or proteins and may have an association with disease phenotypes. This method uses statistical approaches to identify significantly enriched or depleted groups of genes^25^. KEGG pathways and Gene Ontology (GO) enrichment analyses were created for VaD-related genes using DAVID.

“Biological function” terms of GO were used in the study. They describe participation of genes in processes at different levels, from cells to systems of organs, e.g., “positive regulation of apoptosis”, “regulation of blood pressure”, “molecular function” terms describe elementary activities of gene products at molecular level, e.g., ATP binding, kinase activity, “cellular component” terms describe localization of protein in the cell or extracellular environment, e.g., nucleus, plasma membrane. The ontologies have hierarchical structure, from the most common to the most specific terms, and can be presented as directed acyclic graph. The first levels of hierarchy are usually too common and do not provide any useful information, thus, in the current study, we used only terms beginning from third level.

DAVID performs enrichment analysis and allows revealing pathways and GO terms, which are “enriched” with genes from the selected list. To perform an analysis for VaD-related genes, we used “functional annotation” tool with default background gene set and threshold of 0.05 for the Benjamini-Hochberg corrected p-values. Since enriched GO biological processes were redundant, we performed clustering of corresponding terms based on overlaps in their genes. Finally, we manually merged all GO terms from clusters into more general processes reflecting VaD etiopathogenesis.

The PROTEOME database also contains a tool for disease enrichment analysis for sets of genes, which was used in the study.

### DIAMOnD algorithm

It has been shown that functionally related proteins form dense communities in a protein-protein interaction network (i.e., network modules). However, proteins associated with diseases usually do not form dense modules, although they do tend to have immediate connections with each other or through a few neighbours. Therefore, disease modules exist in protein-protein interaction networks, but they are not as dense as functional modules. The DIAMOnD algorithm^22^ designed by Ghiassian and colleagues was used to identify network modules associated with VaD. This algorithm is based on estimating the significance of protein connections and includes four main steps: (1) calculation of the significance of connection of proteins in network with known VaD-related proteins using hypergeometric test; (2) ranking of proteins according to their respective p-values; (3) the protein with the highest rank and correspondingly the lowest p-value is added to the list of known VaD-related proteins; (4) the steps 1–3 are repeated, pulling in one protein at a time into the growing disease module until all network proteins are ranked. The rank of the protein, reflecting its possible relationship to disease, is simply an iteration of the algorithm, when the protein was included into the growing module. We chose DIAMOnD algorithm for the identification of new VaD-related genes, because it allows identifying disease modules rather than functional modules, which contain fewer hubs. This is important because drug action on hubs is associated with significant toxicity, thus, they cannot be selected as potential anti-VaD targets.

We chose the network that was used in a corresponding publication^22^ because it not only contains protein-protein interactions, but also includes direct and indirect interactions of other types: (1) regulatory interactions, which reflect the presence of transcription factor binding sites in the promoters of genes; (2) co-localization of two proteins in protein complexes; (3) metabolic enzyme-coupled interactions, where two enzymes are assumed to be coupled if they share adjacent reactions in the metabolic pathways; (4) signaling interactions, which are the interactions between proteins from signaling pathways; (5) kinase-substrate pairs, which reflect the interactions between kinases and their substrates required for phosphorylation reactions. This network included 13,460 proteins and 141,296 interactions (i.e., protein-protein interactions, protein-DNA regulatory interactions and indirect interactions from metabolic pathways).

### PASS software

The algorithm of PASS is based on atom-centric sub-structural MNA (Multilevel Neighbourhoods of Atoms) descriptors^26^ for structure representation and a Bayesian-like approach for creation of “structure-activity” relationships based on a training set^24^. The prediction results of PASS for a structure include a list of activities along with the probability of each activity being active (Pa) or inactive (Pi). PASS has a 25-year history of use for prediction of biological activity and more than 150 publications with independent experimental confirmation of prediction results, including studies of natural compounds^27^. The online version of PASS is freely available for prediction of single structures (http://www.way2drug.com/PASSOnline/)^28^. PASS 2014 version was used in this study for predicting interactions between phytomolecules and targets associated with VaD. PASS provides predictions for more than 7100 types of biological activities including more than 3500 mechanisms of action (MOAs). This version was created based on a training set with data on more than 900,000 structures of molecules with known biological activity and an average accuracy (AUC) of 0.95 calculated using a leave-one out cross-validation procedure. In this study, PASS was used for a reverse pharmacology assessment of interactions between the studied phytomolecules and select targets related to VaD. Unlike other computational tools predicting interactions of compounds with protein targets (ChemProt^29^, SuperPred^30^, SEA^31^, SwissTargetPrediction^32^, TargetHunter^33^ and DRAR-CPI^34^), PASS predicts types of action of ligand on protein targets (e.g. activation or inhibition). It is very important for the analysis of possible role of the selected targets in pathogenesis of diseases and further planning of experimental studies. In 2018, Murtazalieva with co-authors published the article where showed advantages of PASS software in comparison with ChemProt, SuperPred, SEA, SwissTargetPrediction and TargetHunter^35^.

## Results

### Selection of genes associated with vascular dementia

The list of genes associated with VaD was created based on the data from databases with gene-disease relationships mentioned in the Methods section. During the creation of this list, the associations derived from text-mining results were manually checked by reading the corresponding publications. As a result, a list of 122 genes related to different types of VaD was obtained. Data from humans, rats and mice were evaluated in the analysis. Most of the gene relationships included changes in gene expression for VaD (72 genes) and gene polymorphisms (31 genes). Pharmacological activation or inhibition of 16 proteins is associated with improvements in VaD. From 122 VaD-related genes, 101 had data pointing to a correlation with VaD. The detailed descriptions of these genes are provided in the Supplements section (Table S1). Only 21 genes have more direct relationships: alterations in 4 genes cause VaD, and actions related to 17 genes may prevent VaD (Table 1). These genes may be considered as potential drug targets.

**Table 1:** Twenty one genes associated with vascular dementia.

Name of protein	Gene	Type of VaD	Sp	Alteration	Relationship to VaD	Ref.
Collagen alpha-1(IV) chain	COL4A1	VaD	h	mutation	causes VaD	^61^
Methylenetetrahydrofolate reductase	MTHFR	VaD	h	polymorphism	causes VaD	^62–64^
Neurogenic locus notch homologue protein 3	NOTCH3	CADASIL	h	abnormal folding; absence; decreased processing; mutation	causes VaD	^65–70^
Serine protease HTRA1	HTRA1	CADASIL	h	mutation	causes VaD	^71^
5-hydroxytryptamine receptor 1 A	HTR1A	VaD	h	activation	may prevent VaD	^72^
Acetylcholinesterase	ACHE	VaD	h	inhibition	may prevent VaD	^73^
Alpha-1A adrenergic receptor	ADRA1A	VaD	h	inhibition	may prevent VaD	^74^
Brain-derived neurotrophic factor	BDNF	VaD	r	↑ expression	may prevent VaD	^75^
Cholinesterase	BCHE	Subcortical VaD	h	inhibition	may prevent VaD	^73^
Gamma-aminobutyric acid type B receptor subunit 2	GABBR2	VaD	r	activation	may prevent VaD	^76^
Indoleamine 2,3-dioxygenase 1	IDO1	VaD	r, m	inhibition	may prevent VaD	^56^
Indoleamine 2,3-dioxygenase 2	IDO2	VaD	r, m	inhibition	may prevent VaD	^56^
Kynureninase	KYNU	VaD	r, m	inhibition	may prevent VaD	^56^
Kynurenine/alpha-aminoadipate aminotransferase, mitochondrial	AADAT	VaD	r, m	inhibition	may prevent VaD	^56^
Kynurenine 3-monooxygenase	KMO	VaD	r, m	inhibition	may prevent VaD	^56^
Peroxisome proliferator-activated receptor gamma	PPARG	VaD	h	activation	may prevent VaD	^77,78^
Substance-P receptor	TACR1	VaD	r	inhibition	may prevent VaD	^78^
Tryptophan 2,3-dioxygenase	TDO2	VaD	r, m	inhibition	may prevent VaD	^56^
Type-2 angiotensin II receptor	AGTR2	VaD	m	activation	may prevent VaD	^79^
Sodium-dependent noradrenaline transporter	SLC6A2	VaD	m	inhibition	may prevent VaD	^80^
Aryl hydrocarbon receptor	AHR	VaD	r, m	inhibition	may prevent VaD	^56,81^

Sp - species: h – human, r – rat, m – mouse.

### Identification of VaD-related pathways and biological processes

Enrichment analysis with Gene Ontology (GO) biological processes was conducted with the DAVID service based on the list of 122 selected genes related to VaD. Because GO has several levels of terms, all of the resulting GO biological processes were merged into 15 general processes associated with VaD according to the results of a literature analysis. Names, descriptions of the relationship to VaD and PubMed IDs (identifier of records in PubMed) for each selected GO biological process are shown in Table S2.

Enrichment analysis with KEGG pathways was performed and the VaD-related genes in the resulting pathways were manually analyzed. It allowed us to understand the relationships between KEGG pathways and VaD-related biological processes (Table S3). For example, the PI3K-AKT signaling pathway includes 20 VaD-related genes and the activity of most of those genes were decreased (Table 1, Table S1). The PI3K-AKT pathway map in Fig. S1 shows that VaD-related genes regulate processes associated with cell survival, angiogenesis and protein synthesis. Disruption of these processes contributes to VaD. Thus, we may conclude that the PI3K-AKT pathway is important for pathogenesis of VaD. The complete list of VaD-related KEGG pathways is represented in Table S3.

### Identification of VaD-related diseases

Disease enrichment analysis with 122 VaD-related genes was performed using disease-gene relationships from the PROTEOME database. Many of the revealed disorders were associated with pathogenic mechanisms of VaD, e.g., atherosclerosis, obesity, diabetes, and brain ischemia, whereas other disorders, e.g., Huntington’s and Alzheimer’s diseases, are also neurodegenerative diseases and may share pathogenic mechanisms with VaD. The names and effects of medicinal plants mentioned in Fig. 2 coincide with the treatment of many of these revealed disorders. The number of tumor diseases was also revealed because they share several biological processes with VaD, e.g., cell proliferation, apoptosis, angiogenesis, and autophagy (see Table S2). The top 50 enriched diseases are represented in Table S4. These results confirm that the set of genes associated with VaD was reasonable.

### Identification of drug targets associated with VaD treatment

We collected data on 7 drugs that are used for treating VaD and their targets from the DrugBank and ChEMBL databases. The relationships between 24 drug targets and therapeutic effects were obtained using the existing literature and a database on “mechanism-effect” relationships in PharmaExpert software^36,37^. Only effects that may contribute to improvement of VaD were selected, e.g., nootropic or anti-atherosclerotic effects. Table S5 shows drug-target relationships that may contribute to VaD treatment for known drugs. Twenty nine genes coding revealed targets were added to the list of VaD-related genes. As a result, the final list of VaD-related genes included 147 genes.

### Prediction of VaD-related proteins through a network-based approach

The DIAMOnD algorithm was used for identification of new drug targets based on known target-disease associations and the network interactions to score all proteins in the network (see Materials and Methods). Therefore, it needs to determine a threshold for selecting proteins that are significantly important. The threshold was recommended by Ghiassian and colleagues^22^. We extracted two lists of genes from VaD-related GO biological processes and KEGG pathways (Tables S2 and S3). We took into account only biological processes beginning from third level of GO hierarchy, because first two levels are too general that may introduce a significant bias into results of analysis. The obtained lists contain 1679 and 3624 genes (after excluding VaD-related genes), respectively, that are functionally similar to VaD-related genes. The percentages of these functionally similar genes, which were included in a growing module at each iteration of the algorithm, were calculated (Fig. S2). Both curves were saturated after the 600^th^ iteration. Additionally, we performed similar analysis based on 5-fold cross-validation procedure: one-fifth of 147 VaD-related genes were excluded repeatedly as test sets, and the percentages of genes included in a growing module were calculated. The corresponding curves were also saturated after the 600^th^ iteration (Fig. S2). Therefore, we choose 600 predicted proteins as a threshold for the VaD-related module. Thus, the obtained VaD-related module contained 747 proteins, including 147 known VaD-related proteins as well as 600 predicted new proteins (see Fig. S3). The largest connected component contains 722 proteins. Unfortunately, 5 known VaD-related proteins were not represented in the network and they were not included in the analysis. All 600 new predicted VaD-related proteins along with the algorithm’s ranks are presented in Data file S1 of the Supplement Materials. One can use VaD-related module for analysis and revealing new perspective targets for the treatment of vascular dimension by Cytoscape. We provide the file (VaD-module.xgmml) in Supplement Materials, which can be loaded in Cytoscape^38^.

Since many known relationships between 147 genes and VaD were determined in rats and mice (see Tables 1, S1 and S5), it may cause false discoveries inflation during VaD module construction because a significant fraction of corresponding associations may be species-specific. To estimate the influence of VaD-gene relationships determined in rodents on DIAMOnD prediction results, we excluded corresponding proteins from the list of 147 proteins and re-estimated VaD-related module. We found that 598 out of 600 predicted proteins were observed in re-estimated module, and Spearman correlation coefficient between initial and recalculated ranks was 0.94 with p-value < 10^−16^. We concluded that the presence of genes, whose relationships to VaD were determined in rodents, did not influence the obtained results.

### Identification of significantly overlapped pathways with the list of predicted VaD-related proteins

We selected KEGG pathways which are associated with the list of 600 predicted VaD-related proteins according to two criteria: (1) a pathway contain at least 30 percentages of the proteins from the list and (2) average “600-rank” value of predicted VaD-related proteins in a pathway is at least 300. These thresholds were selected empirically based on the manual analysis of the obtained pathways and their possible relationships to VaD. According to the performed analysis, the pathways below the selected thresholds were usually not related to VaD. As a result a list of fourteen pathways, which is not intersected with the list of pathways from Table S3, was obtained (Table S6). Some of pathways have known associations to VaD, e.g. FoxO signaling pathway, Long-term potentiation and VEGF signaling pathway, whereas others, e.g. Fc gamma R-mediated phagocytosis and Prolactin signaling pathway, are new. The first of two new pathways starts with immunoglobulin receptors and IgG Fc receptor I with rank 275 among 600 predicted VaD-related proteins (see Data file S1). There is no evidence about its involvement into VaD pathogenesis; however the receptor is expressed on microglia and involved into Alzheimer’s pathology as well as up-regulated after ischemic stroke^39,40^. The second pathway starts from prolactin receptor with DIAMOnD rank 272, but the only evidence of its participation in VaD is that the receptor is expressed in astrocytes and activated by ischemia^41^. The discovery of potential links between Fc gamma R-mediated phagocytosis and Prolactin signaling pathway, and VaD may be a basis for further studies.

### Identification of known VaD-related targets interacting with phytomolecules according to PASS prediction

We analyzed the list of 122 genes associated with VaD and selected 55 genes either associated with prevention of VaD (21 genes from Table 1) or correlated with VaD due to an increase in expression, activity or phosphorylation (34 genes from Table S1 with the appropriate description in the Alteration column). The additional 34 genes were selected to extend the field of the search. It was done because in most cases, blocking the action of proteins is easier than activating them from a pharmacological point of view. Thirty-four mechanisms of action (MOAs) predicted by PASS software (see Materials and Methods), which were related to actions conducted on the proteins of 29 genes, were selected. The names of the MOAs, number of active compounds and accuracy of their prediction are shown in Table S7. The predictions of these MOAs were made for the set of structures of 1972 phytomolecules. Only 8 MOAs were predicted for at least one phytomolecule with a probability Pa (Pa means probability to be active, see Materials and Methods) of more than 0.5:

1. Acetylcholinesterase inhibitor – 11 molecules;
2. Adrenaline uptake inhibitor – 6 molecules;
3. Butyrylcholinesterase inhibitor – 5 molecules;
4. Excitatory amino acid transporter 2 inhibitor – 2 molecules;
5. Plasminogen activator inhibitor antagonist – 9 molecules;
6. Tumour necrosis factor antagonist – 14 molecules;
7. Indoleamine-pyrrole 2,3-dioxygenase inhibitor – 10 molecules;
8. Kynureninase inhibitor – 26 molecules.

Based on PASS prediction results, we also selected the plant (Physostigma venenosum) with four phytomolecules (eseridine, physostigmine, physovenine, and eseramine) for which dual MOAs (inhibition of acetylcholinesterase and butyrylcholinesterase) and pharmacological effects related to the treatment of VaD (cognition disorder treatment and nootropic) were predicted with a probability Pa of more than 0.5 (Table S8). Eseridine is an inhibitor of acetylcholinesterase and butyrylcholinesterase^42^. Physostigmine has patents related to inhibition of acetylcholinesterase and the treatment of AD (EP0296560), enhancing memory (US5177101, US2007197663), inhibition of butyrylcholinesterase and treatment of Alzheimer’s disease as well as dementia (EP0949920). Physovenine was patented as an inhibitor of acetylcholinesterase and butyrylcholinesterase as well as for the treatment of AD and dementia (EP0949920, WO9902154). Eseramine has less inhibitory activity against acetylcholinesterase compared to physostigmine with IC_50_ values of 9300 nM and 61 nM, respectively^43^. The presence of several active phytomolecules against acetylcholinesterase and butyrylcholinesterase may cause additive or synergistic effects of Physostigma venenosum in the treatment of VaD. Currently, it is known that this plant is used for the treatment of atherosclerosis (Data file S2).

### Identification of new VaD-related targets interacting with phytomolecules according to PASS predictions

Six hundred genes is a rather large number and does not account for all proteins that are the products of these genes, although experimental data about known active and inactive compounds exist. Moreover, it is necessary to know how drugs should act on a target and whether they stimulate or inhibit its activity. We selected 175 genes from 600 genes, of which 178 related mechanisms of action were predicted by PASS. The mean accuracy (AUC value) of prediction calculated by a leave-one out cross-validation procedure was 0.969. The names of the MOAs, number of active compounds and AUC are shown in Table S9. Predictions of 178 MOAs were made for 1972 phytomolecules by PASS. The analysis of prediction results showed that only 25 MOAs were predicted for 5 or more phytomolecules with a probability Pa of more than 0.5. The literature and Cytoscape analysis of these 25 MOAs and MOAs related to the top 50 from 600 selected targets and predicted by PASS with Pa value less than 0.5 revealed 10 MOAs coinciding with potential treatment of VaD (Table 2). Table 2 shows also known approved drugs acting on the selected targets according to data from DrugBank^16^. These 24 drugs may be studied as drugs for the treatment of VaD and serve the basis of their repositioning.

**Table 2:** Ten selected mechanisms of action related to the actions of phytomolecules on new targets, which may be used for the treatment of VaD.

Mechanism	UniProt ID	Rank	Effects	Reference	DrugBank
Nitric oxide synthase, brain inhibitor	P29475	9	Neuroprotection, reducing oxidative stress in the brain, anti-depression, and anti-anxiety.	^82–86^	Ketamine; Methylene blue
Glycogen synthase kinase-3 alpha inhibitor	P49840	13	cognitive improvement, anti-diabetic, vasoprotective, Alzheimer’s treatment, bipolar disorder treatment	^87–90^	Fostamatinib
Bradykinin B2 receptor antagonist	P30411	15	Reduces ischaemic infarct volume, reduces post-ischaemic neuronal oedema, improves neuronal function recovery, reduces blood brain barrier disruption, protects memory deficits, angioedema treatment, blocks propagation of inflammation.	^50,51,91–93^	Icatibant
Extracellular calcium sensing receptor antagonist	P41180	36	Neuroprotection from traumatic and ischaemic brain injury, blood pressure regulation, vascular tone regulation, regulation of synaptic plasticity, central control of systemic fluid and electrolytes, regulation of cytokine, NO and ROS production.	^94–98^	—
Alpha 2 A adrenergic receptor antagonists	P08913	49	Increases special memory acquisition, learning and memory; potentiates inhibition of acetylcholinesterase; anti-diabetic (increases insulin secretion; reduces blood glucose).	^99–109^	Phentolamine; Mirtazapine; Yohimbine; Phenoxybenzamine; Propericiazine
Cystic fibrosis transmembrane conductance regulator agonist	P13569	104	Neuroprotection, increases insulin secretion.	^110–113^	Bumetanide; Crofelemer; Ibuprofen; Dexibuprofen
Insulin-like growth factor 1 receptor agonist	P08069	109	Neuroprotection, anti-diabetic, dual action on atherosclerosis, hypotensive effect.	^114–122^	Mecasermin
Androgen receptor agonist	P10275	289	Dual effects on cerebral ischaemia consequences, atherosclerosis treatment, anti-diabetic, anti-obesity, promotion of angiogenesis.	^123–134^	Oxandrolone; Testosterone; Nandrolone phenpropionate; Fluoxymesterone; Danazol; Nandrolone decanoate; Methyltestosterone; Oxymetholone
Glycogen synthase kinase-3 beta inhibitor	P49841	296	Neuroprotectant, cognitive improvement, anti-diabetic, vasoprotective, Alzheimer’s treatment	^87,88,135^	Fostamatinib
Protein phosphatase 1B inhibitor	P18031	507	Angiogenesis promotion (anti-ischaemic effect), anti-diabetic, dual action on atherosclerosis, anti-obesity.	^48,49,136–139^	Tiludronic acid

UniProt ID – identifier of protein in UniProt database, which is related to the appropriate mechanism of action; DrugBank – approved drugs with the appropriate mechanism of action from DrugBank database.

## Discussion

**Figure 3:**
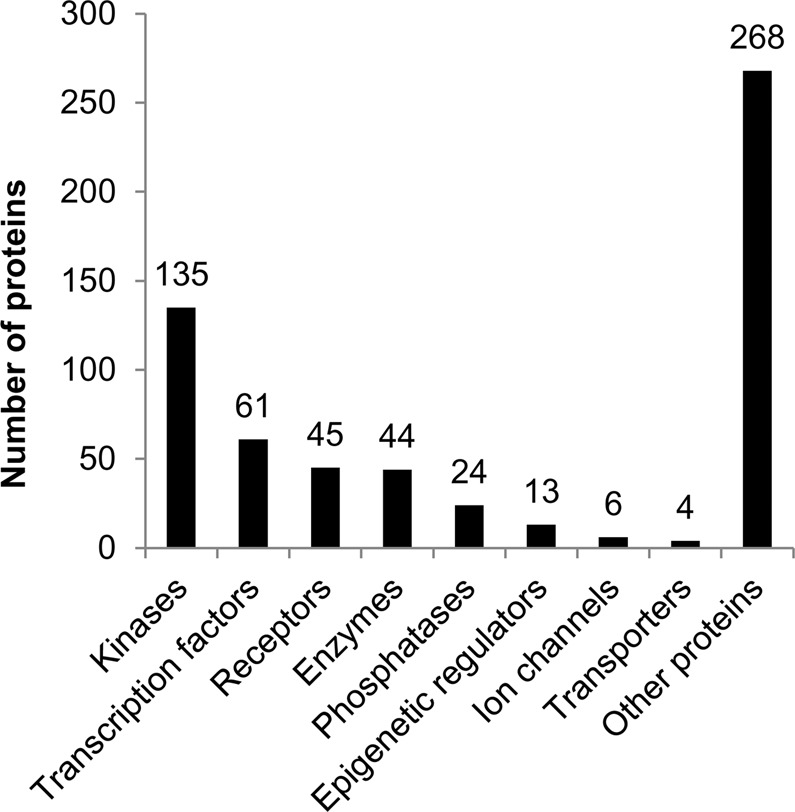
Distribution of new targets according to classes of proteins.

During the analysis of the literature and databases, including data on the relationships between genes and diseases, we found 147 genes associated with VaD. Some proteins of these genes (e.g., acetylcholinesterase) are used as drug targets for the treatment of dementia. The proteins related to other genes may also be studied as potential drug targets for the treatment of VaD, especially the proteins mentioned in Table 1. Using systems biology and network pharmacology methods, we found 600 new potential drug targets that may be related to the treatment of VaD. These targets belong to different types of proteins according to the protein classification used in ChEMBL database (Fig. 3).

Most of these proteins belong to kinases. The search for compounds, which includes selective inhibitors of a kinase, is a rather difficult task. Therefore, not all of the selected targets may be convenient for searching for drug candidates. From 600 selected genes, only 370 protein targets related to them had records in ChEMBL with data on testing compounds (Data file S1). The ChEMBL 22 database includes a list of drugs that interact with 106 targets related to the selected genes. All of these targets may be considered druggable^44^. Data file S1 includes information about the targets interacting with 692 known drugs in different stages of drug development. The names of 692 drugs interacting with 106 protein-targets related to the selected genes are represented in Data file S3. These data may be used for repurposing known drugs.

The analysis of Protein Data Bank (PDB, https://www.rcsb.org) revealed 473 proteins that have at least one PDB record with 3D structures (Data file S1). Many of these targets may also be considered potential drug targets because the knowledge of 3D structures of the proteins promotes finding new active compounds^45^. Currently, only 36 targets have PDB records, including complexes between drugs and proteins with more than 70% of protein sequence identity. This estimation was made based on the analysis of data from the “Drug and Drug Target Mapping” section of the PDB (http://www.rcsb.org/pdb/ligand/drugMapping.do).

The information on the type of drug action on targets is necessary in the search for new drugs. To reduce the number of targets that need to be analyzed to reveal the type of drug action, we found proteins that interact with the studied phytomolecules. As a result, the 10 most promising mechanisms of action and appropriate targets were selected. These targets and MOAs should be studied further in the search for phytomolecules related to the treatment of vascular dementia. Our study also revealed possible relation between Fc gamma R-mediated phagocytosis and Prolactin signaling pathways and VaD that may be a basis for further studies.

**Figure 4:**
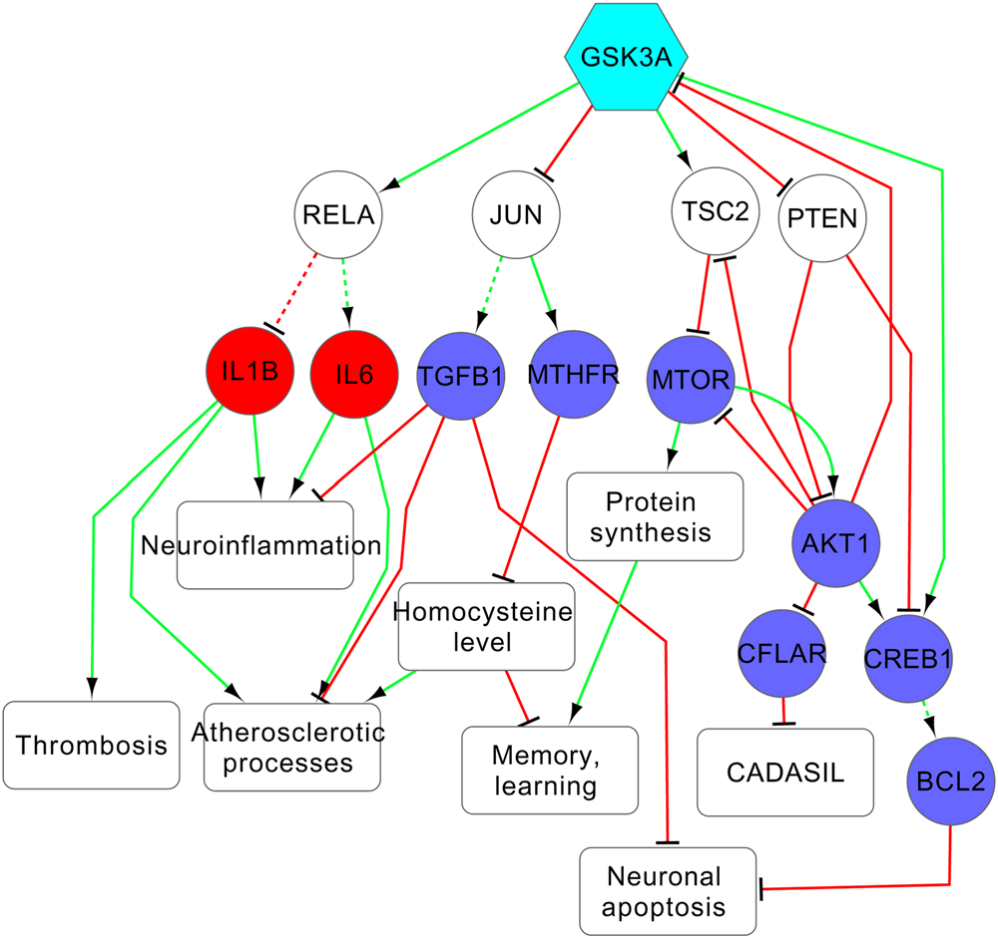
Mechanisms related to possible therapeutic effect of glycogen synthase kinase-3 alpha (GSK3a) inhibition. The red and blue colors of nodes represent the known information about the direction of changing of protein function or expression in VaD: increasing or decreasing, correspondingly. The cyan color of nodes represents proteins, whose relationships with VaD were predicted by DIAMOnD algorithm.

**Figure 5:**
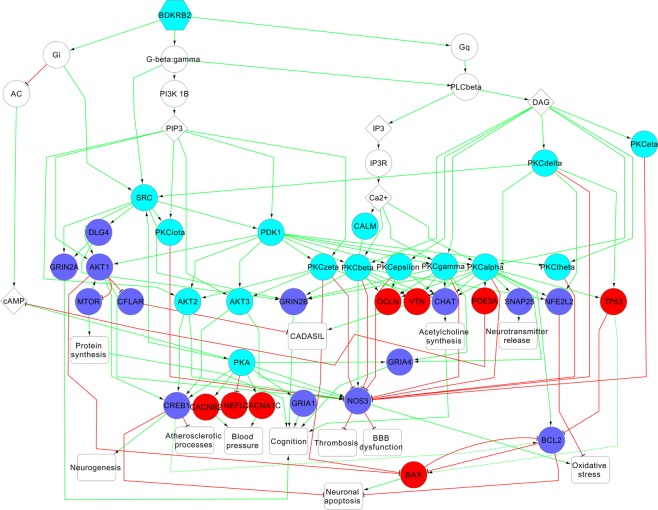
Mechanisms related to possible therapeutic effect of bradykinin B2 receptor (BDKRB2) antagonism. The red and blue colors of nodes represent the known information about the direction of changing of protein function or expression in VaD: increasing or decreasing, correspondingly. The cyan color of nodes represents proteins, whose relationships with VaD were predicted by DIAMOnD algorithm.

**Figure 6:**
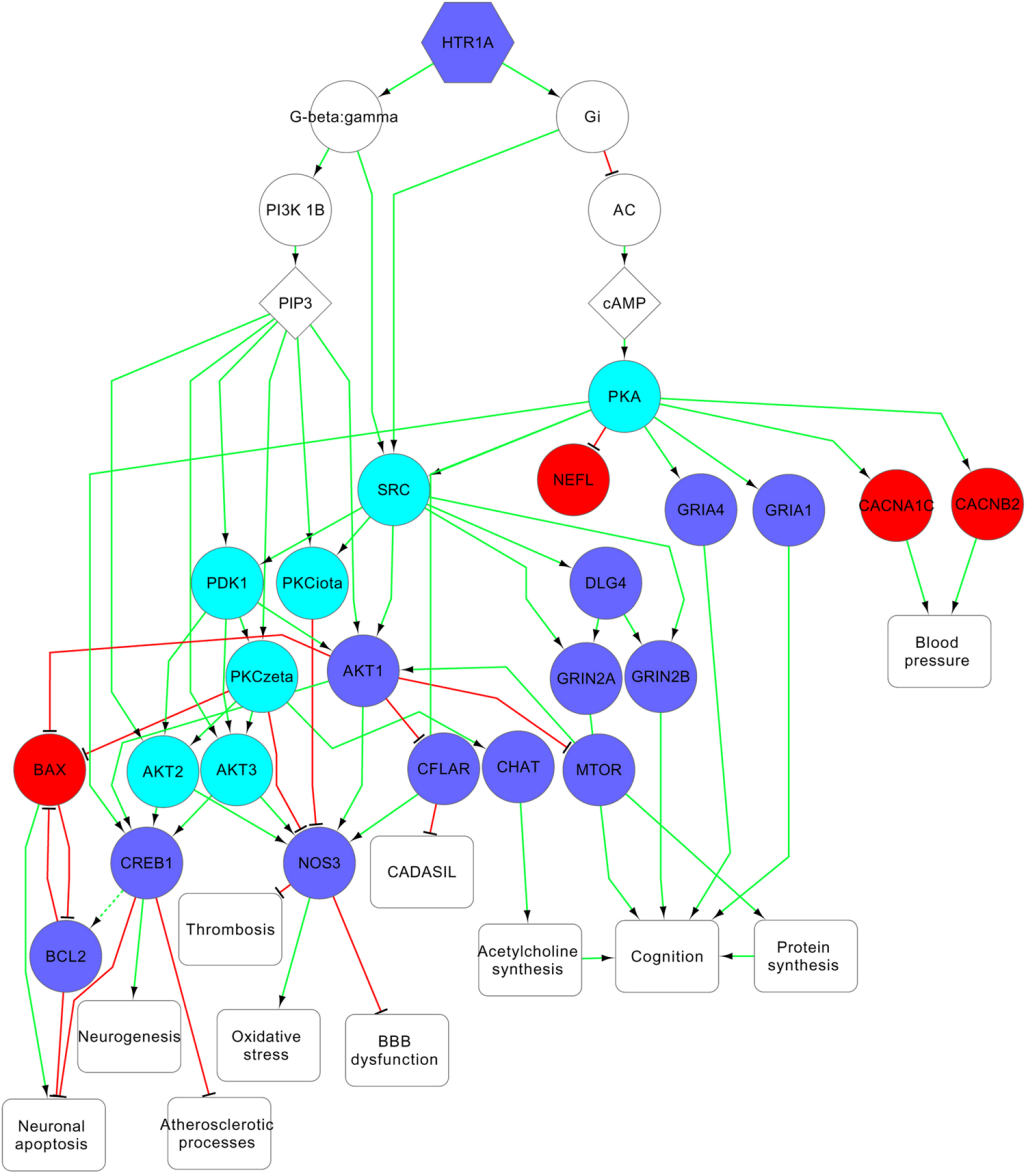
Mechanisms related to possible therapeutic effect of 5-hydroxytryptamine receptor 1A (HTR1A) activation. The red and blue colors of nodes represent the known information about the direction of changing of protein function or expression in VaD: increasing or decreasing, correspondingly. The cyan color of nodes represents proteins, whose relationships with VaD were predicted by DIAMOnD algorithm.

The given results may be used for deeper analysis of networks explaining relations between selected targets and pathogenesis of vascular dementia. To display it we made such analysis for 3 new VaD-related targets from Table 2 (Glycogen synthase kinase-3 alpha; Protein phosphatase 1B and Bradykinin B2 receptor) and two targets with known associations to vascular dementia, which are not used for the treatment of vascular dementia at the present time, from Table 1 (Indoleamine 2,3-dioxygenase and 5-hydroxytryptamine receptor 1A) based on information about directed interactions between proteins from SIGNOR 2.0 database^23^. Activating and inhibiting as types of interactions were used in the study. We calculated shortest paths from the appropriate target to proteins with known relationships to VaD and selected the paths with the appropriate targets interacting with VaD-related proteins directly or through one intermediate protein. All interactions between proteins of selected shortest paths were extracted. The relationships between proteins with known VaD-associations and corresponding pathological processes leading to the disease were found based on analysis of literature. In the Figs. 4–6, red edges are inhibiting interactions, green edges are activating interactions, blue nodes are proteins whose expression/function is decreased in vascular dementia, red nodes are proteins whose expression/function is increased in vascular dementia, solid and dotted lines are direct or indirect interactions through transcription regulation, respectively. The Figs. 4 and 5 shows mechanisms related to possible therapeutic effect of the inhibition of glycogen synthase kinase-3 alpha (GSK3A) and bradykinin B2 receptor (BDKRB2).

The therapeutic effect possibly related to the influence on processes taking place in the brain during ischemia, such as neuronal inflammation and apoptosis, oxidative stress, decrease in protein synthesis and neurotransmitter release, disruption of blood-brain barrier, and increase in angiogenesis. The modulation of these processes may cause neuroprotective effect and increase cognitive ability. The inhibition of GSK3A, PTP1B and BDKRB2 may be also associated with modulation of VaD risk factors, such as atherosclerosis and hypertension. The relationships between inhibition of three potential targets and influence on some brain related processes, as well as risk factors, were confirmed by analysis of the literature. Deficiency of GSK3A is known to be associated with enhanced insulin sensitivity, glucose tolerance and attenuation of atherosclerotic processes^46^. The mutation in the corresponding gene affects long-term potentiation and depression; thus, inhibition of this kinase may be used to treat cognitive disorders^47^. Decrease in function of PTP1B is known to promote angiogenesis and attenuate atherosclerotic processes^48,49^. Antagonists of BDKRB2 reduce ischemic infarct volume, blood brain barrier disruption and oxidative stress, improve neuronal function recovery and protect from memory deficits^50,51^. Knockout of BDKRB2 gene is also associated with decreased thrombosis^52^.

The Fig. 6 shows mechanisms related to possible therapeutic effect of 5-hydroxytryptamine receptor 1 A (HTR1A) activation. Tandospirone, a partial agonist of HTR1A, is effective in the treatment of behavioral and psychological symptoms associated with dementia^53^. The activation of HTR1A is associated with neuroprotective, anti-inflammatory and anti-oxidative effects, as well as preventing of permeability changes in blood-brain barrier^54,55^.

**Figure 7:**
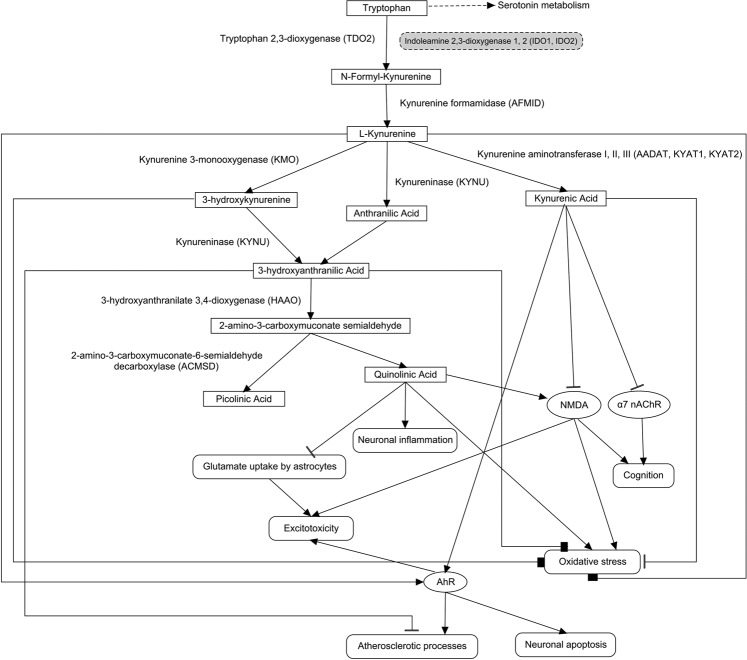
Mechanisms related to possible therapeutic effect of indoleamine 2,3-dioxygenase (IDO) inhibition. The grey color of node highlights indoleamine 2,3-dioxygenases.

The Fig. 7 shows mechanisms related to possible therapeutic effect of indoleamine 2,3-dioxygenase 1 (IDO1) inhibition. The IDO1 is one of the most important enzymes of kynurenine pathway, whose metabolites play important role in brain ischemia and VaD^56^. The decrease in function of IDO1 is known to be associated with protection against NMDA receptor-mediated excitotoxicity^57^ and atherosclerosis^58^.

These targets may guide wet lab experimentations or can be targeted directly by therapies (concept of repurposing). To confirm relations of selected targets with the treatment of VaD we made *in vivo* validation of two selected targets on mice. The experimental results of these studies were recently reported in our others publications^59,60^. Inhibition of glycogen synthase kinase-3 was studied using lithium carbonate, a classical inhibitor of glycogen synthase kinase-3 (GSK-3), on the mice model of mild chronic cerebral hypoperfusion^59^. This study confirmed relation between inhibition of GSK-3 and the treatment of VaD. It was shown that lithium carbonate reduces memory impairments, emotion and recognition, spatial and fear-motivated learning along with diminution glutamate-induced excitotoxicity in cerebral cortex and hippocampus, cholinergic dysfunction and oxidative stress.^59^. The second selected target was protein tyrosine phosphatase 1B (PTP1B). The relation of PTP1B with VaD was studied by inhibition of sodium orthovanadate, on mice with homocysteine-induced endothelial dysfunction, cholinergic learning dysfunctionm, oxidative stress and memory impairments^60^. It was shown that sodium orthovanadate significantly improved memory impairment, the emotional and fear-motivated learning of mice^60^. Based on these studies we hope that others selected targets will also demonstrate relations with the treatment of vascular dementia.

## Conclusions

Our results showed that 10 new most possible drug targets related with the treatment of vascular dementia were selected based on using the combination of network pharmacology and reverse pharmacology approaches. Starting from the analysis of know drugs used for the treatment of symptoms of VaD and known associations between genes and VaD using gene set enrichment analysis and DIAMOnD algorithm, 600 new possible drug targets associated with the treatment of vascular dementia were selected. Because of the large amount of new possible drug targets were found, we estimated possible interaction between known phytocomponents of medicinal plants used for the treatment of VaD and related diseases by PASS prediction. It considerably reduced the number of potential VaD-related drug targets and displayed the way for the search of new medicinal plants which may be used for the treatment of VaD. Additional analysis between of selected targets and pathogenesis of vascular dementia based on the data from SIGNOR 2.0 database showed validity the selected targets and their influence on pathogenesis of VaD. Experimental studies of known inhibitors of two selected targets (glycogen synthase kinase-3 and protein tyrosine phosphatase 1B) showed their relation with the treatment of VaD on animal models. Others three targets (bradykinin B2 receptor, 5-hydroxytryptamine receptor 1A and indoleamine 2,3-dioxygenase 1) are under the study. The analysis of FDA approved drugs showed that some of them interact with some new identified VaD-related drug targets. It may be used as a basis for the experimental and clinical studies directed on repurposing the appropriate drugs for the treatment of vascular dementia.

## Supplementary information

Supplementary Information 

.Supplementary Information2

Supplementary Information3

Supplementary Information4

Supplementary Information5

Supplementary Information6

## References

[1] Livingston G (2017). Dementia burden coming into focus. Lancet.

[2] Thal DR, Grinberg LT, Attems J (2012). Vascular dementia: different forms of vessel disorders contribute to the development of dementia in the elderly brain. Exp. Geronto..

[3] Budson, A. E. & Solomon, P. R. Chapter 6 – Vascular Dementia and Vascular Cognitive Impairment, in: Memory Loss, Alzheimer’s Disease, and Dementia. In: A Practical Guide for Clinicians. 2nd ed. 80–89 (Elsevier, 2016).

[4] Gomazkov OA, Lagunin AA (2017). Vascular Dementia: Molecular Targets of Neuroprotective Therapy. Biol. Bull. Rev..

[5] Albert M (2017). Heart risks in middle age boost dementia risk later in life. Am. Stroke Assoc. Meet. Report, Session A.

[6] Kling MA, Trojanowski JQ, Wolk DA, Lee VM, Arnold SE (2013). Vascular disease and dementias: paradigm shifts to drive research in new directions. Alzheimers Dement..

[7] Vidal M, Cusick ME, Barabási A-L (2011). Interactome Networks and Human Disease. Cell.

[8] Caldera M, Buphamalai P, Müller F, Menche J (2017). Interactome-based approaches to human disease. Curr Opin. Syst. Biol..

[9] Zhao J, Jiang P, Zhang W (2010). Molecular networks for the study of TCM Pharmacology. Brief. Bioinform.

[10] Li Jian, Lu Cheng, Jiang Miao, Niu Xuyan, Guo Hongtao, Li Li, Bian Zhaoxiang, Lin Na, Lu Aiping (2012). Traditional Chinese Medicine-Based Network Pharmacology Could Lead to New Multicompound Drug Discovery. Evidence-Based Complementary and Alternative Medicine.

[11] Tao WY (2013). Network pharmacology-based prediction of the active ingredients and potential targets of Chinese herbal radix curcumae formula for application to cardiovascular disease. J. Ethnopharmacol..

[12] Li S, Zhang B (2013). Traditional Chinese medicine network pharmacology: theory, methodology and application. Chin. J. Nat. Med..

[13] Piñero J (2017). DisGeNET: a comprehensive platform integrating infor-mation on human disease-associated genes and variants. Nucleic Acids Res..

[14] Liu CC (2014). DiseaseConnect: a comprehensive web server for mecha-nism-based disease-disease connections. Nucleic Acids Res..

[15] Pletscher-Frankild S, Pallejà A, Tsafou K, Binder JX, Jensen LJ (2015). DISEASES: text mining and data integration of disease-gene associations. Methods.

[16] Wishart DS (2018). DrugBank 5.0: a major update to the DrugBank database for 2018. Nucleic Acids Res..

[17] Gaulton A (2017). The ChEMBL database in 2017. Nucleic Acids Res..

[18] Wang Y (2017). PubChem BioAssay: 2017 update. Nucleic Acids Res..

[19] Kanehisa M, Sato Y, Furumichi M, Morishima K, Tanabe M (2019). New approach for understanding genome variations in KEGG. Nucleic Acids Res..

[20] The Gene Ontology Consortium (2019). The Gene Ontology Resource: 20 years and still GOing strong. Nucleic Acids Res..

[21] Sherman BT, Lempicki RA (2009). Bioinformatics enrichment tools: paths toward the comprehensive functional analysis of large gene lists. Nucleic Acids Res..

[22] Ghiassian SD, Menche J, Barabási AL (2015). A DIseAse MOdule Detection (DIAMOnD) algorithm derived from a systematic analysis of connectivity patterns of disease proteins in the human interactome. PLoS Comput. Biol..

[23] Perfetto L (2016). SIGNOR: a database of causal relationships between biological entities. Nucleic Acids Res..

[24] Poroikov VV, Filimonov DA, Borodina YV, Lagunin AA, Kos A (2000). Robustness of biological activity spectra predicting by computer program PASS for non-congeneric sets of chemical compounds. J. Chem. Inf. Comput. Sci..

[25] Subramanian A (2005). Gene set enrichment analysis: a knowledge-based approach for interpreting genome-wide expression profiles. Proc. Natl. Acad. Sci. USA.

[26] Filimonov D, Poroikov V, Borodina Y, Gloriozova T (1999). Chemical Similarity Assessment through Multilevel Neighborhoods of Atoms:  Definition and Comparison with the other descriptors. J. Chem. Inf. Comput. Sci..

[27] Filimonov DA (2014). Prediction of the Biological Activity Spectra of Organic Compounds Using the PASS Online Web Resource. Chem. Heterocycl. Compd..

[28] Lagunin A, Stepanchikova A, Filimonov D, Poroikov V (2000). PASS: Prediction of Activity Spectra for Biologically Active Substances. Bioinformatics.

[29] Kringelum J (2016). ChemProt-3.0: a global chemical biology diseases mapping. Database (Oxford)..

[30] Nickel J (2014). SuperPred: update on drug classification and target prediction. Nucleic Acids Res..

[31] Keiser MJ (2007). Relating protein pharmacology by ligand chemistry. Nat. Biotechnol..

[32] Daina A, Michielin O, Zoete V (2019). SwissTargetPrediction: updated data and new features for efficient prediction of protein targets of small molecules. Nucleic Acids Res..

[33] Wang L (2013). TargetHunter: an *in silico* target identification tool for predicting therapeutic potential of small organic molecules based on chemogenomic database. AAPS J..

[34] Luo H (2011). DRAR-CPI: a server for identifying drug repositioning potential and adverse drug reactions via the chemical-protein interactome. Nucleic Acids Res..

[35] Murtazalieva KA, Druzhilovskiy DS, Goel RK, Sastry GN, Poroikov VV (2017). How good are publicly available web services that predict bioactivity profiles for drug repurposing?. SAR. QSAR Env. Res..

[36] Lagunin A, Filimonov D, Poroikov V (2010). Multi-Targeted Natural Products Evaluation Based on Biological Activity Prediction with PASS. Curr. Pharm. Des..

[37] Lagunin AA (2014). Chemo- and bioinformatics resources for in silico drug discovery from medicinal plants beyond their traditional use: a critical review. Nat. Prod. Rep..

[38] Su G, Morris JH, Demchak B, Bader GD (2014). Biological network exploration with Cytoscape 3. Curr. Protoc. Bioinformatics..

[39] Adamski MG (2014). Expression profile based gene clusters for ischemic stroke detection. Genomics.

[40] Minett T (2016). Microglial immunophenotype in dementia with Alzheimer’s pathology. J. Neuroinflammation.

[41] Möderscheim TA (2007). Prolactin is involved in glial responses following a focal injury to the juvenile rat brain. Neuroscience.

[42] Yu QS (2002). Anticholinesterase activity of compounds related to geneser-ine tautomers. N-Oxides and 1,2-oxazines. J. Med. Chem..

[43] Yu QS, Atack JR, Rapoport SI, Brossi A (1988). Synthesis and anticholinesterase activity of (−)-N1-norphysostigmine, (−)-eseramine, and other N(1)-substituted analogues of (−)-physostigmine. J. Med. Chem..

[44] Al-Lazikani, B. *et al*. The Molecular Basis of Predicting Druggability. In Chemical Biology: From Small Molecules to Systems Biology and Drug Design 1–3. (eds Wess, G, Schreiber, S. L. & Kapoor, T. M.) 804–823 (Wiley-VCH, 2007).

[45] Halgren TA (2009). Identifying and characterizing binding sites and assessing druggability. J. Chem. Inf. Model..

[46] Banko NS (2014). Glycogen synthase kinase 3α deficiency attenuates atherosclerosis and hepatic steatosis in high fat diet-fed low density lipoprotein receptor-deficient mice. Am. J. Pathol..

[47] Shahab L, Plattner F, Irvine EE, Cummings DM, Edwards FA (2014). Dynamic range of GSK3α not GSK3β is essential for bidirectional synaptic plasticity at hippocampal CA3-CA1 synapses. Hippocampus.

[48] Lanahan AA (2014). PTP1b is a physiologic regulator of vascular endothelial growth factor signaling in endothelial cells. Circulation.

[49] Zhou X, Xu W, Chen J (2011). The 981 C > T polymorphism in protein tyrosine phosphatase 1B is associated with decreased risk of coronary artery disease in Chinese Han population. Atherosclerosis.

[50] Ding-Zhou L (2003). LF 16-0687 Ms, a bradykinin B2 receptor antagonist, reduces ischemic brain injury in a murine model of transient focal cerebral ischemia. Br. J. Pharmacol..

[51] Ferreira AP (2014). HOE-140, an antagonist of B2 receptor, protects against memory deficits and brain damage induced by moderate lateral fluid percussion injury in mice. Psychopharmacol. (Berl.).

[52] Fang C (2013). Angiotensin 1–7 and Mas decrease thrombosis in Bdkrb2−/− mice by increasing NO and prostacyclin to reduce platelet spreading and glycoprotein VI activation. Blood.

[53] Huang X (2017). Role of tandospirone, a 5-HT1A receptor partial agonist, in the treatment of central nervous system disorders and the underlying mechanisms. Oncotarget.

[54] Hind. WH, England TJ, O’Sullivan SE (2016). Cannabidiol protects an in vitro model of the blood-brain barrier from oxygen-glucose deprivation via PPARγ and 5-HT1A receptors. Br. J. Pharmacol..

[55] Pazos MR, Mohammed N, Lafuente H (2013). Mechanisms of cannabidiol neuroprotection in hypoxic-ischemic newborn pigs: role of 5HT(1A) and CB2 receptors. Neuropharmacology.

[56] Cuartero MI (2016). The Kynurenine Pathway in the Acute and Chronic Phases of Cerebral Ischemia. Curr. Pharm. Des..

[57] Mazarei G (2013). The absence of indoleamine 2,3-dioxygenase expression protects against NMDA receptor-mediated excitotoxicity in mouse brain. Exp. Neurol..

[58] Yeung AW, Terentis AC, King NJ, Thomas SR (2015). Role of indoleamine 2,3-dioxygenase in health and disease. Clin. Sci. (Lond.).

[59] Kumar S, Ivanov S, Lagunin A, Goel RK (2019). Glycogen synthase kinase-3 inhibition as a potential pharmacological target for vascular dementia: In silico and in vivo evidence. Comput. Biol. Med..

[60] Kumar S, Ivanov S, Lagunin A, Goel RK (2019). Attenuation of hyperhomocysteinemia induced vascular dementia by sodium orthovanadate perhaps via PTP1B: Pertinent downstream outcomes. Behav. Brain Res..

[61] Sibon I (2007). COL4A1 mutation in Axenfeld-Rieger anomaly with leukoencephalopathy and stroke. Ann. Neurol..

[62] Yoo JH, Choi GD, Kang SS (2000). Pathogenicity of thermolabile methylenetetrahydrofolate reductase for vascular dementia. Arterioscler. Thromb. Vasc. Biol..

[63] McIlroy SP, Dynan KB, Lawson JT, Patterson CC, Passmore AP (2002). Moderately elevated plasma homocysteine, methylenetetrahydrofolate reductase genotype, and risk for stroke, vascular dementia, and Alzheimer disease in Northern Ireland. Stroke.

[64] Jin P (2013). Association between MTHFR gene polymorphisms, smoking, and the incidence of vascular dementia. Asia. Pac. J. Public. Health..

[65] Malandrini A (2002). Asymptomatic cores and paracrystalline mitochondrial inclusions in CADASIL. Neurology.

[66] Utku U, Celik Y, Uyguner O, Yüksel-Apak M, Wollnik B (2002). CADASIL syndrome in a large Turkish kindred caused by the R90C mutation in the Notch3 receptor. Eur. J. Neurol..

[67] Ceroni M (2000). Migraine with aura and white matter abnormalities: Notch3 mutation. Neurology.

[68] Liebetrau M, Herzog J, Kloss CU, Hamann GF, Dichgans M (2002). Prolonged cerebral transit time in CADASIL: a transcranial ultrasound study. Stroke.

[69] Joutel A (2000). The ectodomain of the Notch3 receptor accumulates within the cerebrovasculature of CADASIL patients. J. Clin. Invest..

[70] Dichgans M, Herzog J, Gasser T (2001). NOTCH3 mutation involving three cysteine residues in a family with typical CADASIL. Neurology.

[71] Tikka S (2014). CADASIL and CARASIL. Brain Pathol..

[72] Sato S, Mizukami K, Asada T (2007). A preliminary open-label study of 5-HT1A partial agonist tandospirone for behavioural and psychological symptoms associated with dementia. Int. J. Neuropsychopharmacol..

[73] Moretti R, Torre P, Antonello RM, Cazzato G, Bava A (2002). Rivastigmine in subcortical vascular dementia: an open 22-month study. J. Neurol. Sci..

[74] Saletu B, Garg A, Shoeb A (2014). Safety of nicergoline as an agent for management of cognitive function disorders. Biomed. Res. Int..

[75] Sakr HF (2014). Effect of dehydroepiandrosterone (DHEA) on memory and brain derived neurotrophic factor (BDNF) in a rat model of vascular dementia. J. Physiol. Pharmacol..

[76] Li CJ (2014). Activation of GABAB receptors ameliorates cognitive impairment via restoring the balance of HCN1/HCN2 surface expression in the hippocampal CA1 area in rats with chronic cerebral hypoperfusion. Mol. Neurobiol..

[77] Kishi T, Hirooka Y, Sunagawa K (2012). Telmisartan protects against cognitive decline via up-regulation of brain-derived neurotrophic factor/tropomyosin-related kinase B in hippocampus of hypertensive rats. J. Cardiol..

[78] Kaur J, Sharma S, Sandhu M, Sharma S (2016). Neurokinin-1 receptor inhibition reverses ischaemic brain injury and dementia in bilateral common carotid artery occluded rats: possible mechanisms. Inflammopharmacology.

[79] Iwanami J (2015). Direct angiotensin II type 2 receptor stimulation by compound 21 prevents vascular dementia. J. Am. Soc. Hypertens..

[80] Gupta S, Sharma B, Singh P, Sharma BM (2014). Modulation of transient receptor potential vanilloid subtype 1 (TRPV1) and norepinephrine transporters (NET) protect against oxidative stress, cellular injury, and vascular dementia. Curr. Neurovasc Res..

[81] Cuartero MI (2014). L-kynurenine/aryl hydrocarbon receptor pathway mediates brain damage after experimental stroke. Circulation.

[82] Peeters-Scholte C (2002). Neuroprotection by selective nitric oxide synthase inhibition at 24 h after perinatal hypoxia-ischemia. Stroke.

[83] Garry PS, Ezra M, Rowland MJ, Westbrook J, Pattinson KT (2015). The role of the nitric oxide pathway in brain injury and its treatment–from bench to bedside. Exp. Neurol..

[84] Vitcheva V, Simeonova R, Kondeva-Burdina M, Mitcheva M (2015). Selective Nitric Oxide Synthase Inhibitor 7-Nitroindazole Protects against Cocaine-Induced Oxidative Stress in Rat Brain. Oxid. Med. Cell Longev..

[85] Harkin AJ, Bruce KH, Craft B, Paul IA (1999). Nitric oxide synthase inhibitors have antidepressant-like properties in mice. 1. Acute treatments are active in the forced swim test. Eur. J. Pharmacol..

[86] Sevgi S, Ozek M, Eroglu L (2006). L-NAME prevents anxiety-like and depression-like behavior in rats exposed to restraint stress. Methods Find. Exp. Clin. Pharmacol..

[87] Kanzariya NR, Patel RK, Patel NJ (2011). Antidiabetic and vasoprotective activity of lithium: Role of glycogen synthase kinase-3. Indian. J. Pharmacol..

[88] Avila. J, Wandosell F, Hernández F (2010). Role of glycogen synthase kinase-3 in Alzheimer’s disease pathogenesis and glycogen synthase kinase-3 inhibitors. Expert. Rev. Neurother..

[89] Li X, Liu M, Cai Z, Wang G, Li X (2010). Regulation of glycogen synthase kinase-3 during bipolar mania treatment. Bipolar Disord..

[90] Gould TD, Chen G, Manji HK (2004). *In vivo* evidence in the brain for lithium inhibition of glycogen synthase kinase-3. Neuropsychopharmacology.

[91] Kaplanski J (2002). LF 16-0687 Ms, a bradykinin B2 receptor antagonist, reduces brain edema and improves long-term neurological function recovery after closed head trauma in rats. J. Neurotrauma.

[92] Bartal C, Zeldetz V, Stavi V, Barski L (2015). The role of icatibant-the B2 bradykinin receptor antagonist-in life-threatening laryngeal angioedema in the ED. Am. J. Emerg. Med..

[93] Terzuoli E (2014). Antagonism of bradykinin B2 receptor prevents inflammatory responses in human endothelial cells by quenching the NF-kB pathway activation. PLoS One.

[94] Kim JY, Kim N, Yenari MA, Chang W (2013). Hypothermia and pharmacological regimens that prevent overexpression and overactivity of the extracellular calcium-sensing receptor protect neurons against traumatic brain injury. J. Neurotrauma.

[95] Kim JY (2014). Calcium-sensing receptor (CaSR) as a novel target for ischemic neuroprotection. Ann. Clin. Transl. Neurol..

[96] Kobayashi-Torii M (2011). Possible participation of extracellular calcium-sensing receptor in blood pressure regulation in rats. Brain Res..

[97] Schepelmann M (2016). The vascular Ca2 + -sensing receptor regulates blood vessel tone and blood pressure. Am. J. Physiol. Cell Physiol..

[98] Bandyopadhyay S, Tfelt-Hansen J, Chattopadhyay N (2010). Diverse roles of extracellular calcium-sensing receptor in the central nervous system. J. Neurosci. Res..

[99] Gazarini L, Stern CA, Carobrez AP, Bertoglio LJ (2013). Enhanced noradrenergic activity potentiates fear memory consolidation and reconsolidation by differentially recruiting α1- and β-adrenergic receptors. Learn. Mem..

[100] Torkaman-Boutorabi A, Danyali F, Oryan S, Ebrahimi-Ghiri M, Zarrindast MR (2014). Hippocampal α-adrenoceptors involve in the effect of histamine on spatial learning. Physiol. Behav..

[101] Haapalinna A, Sirviö J, MacDonald E, Virtanen R, Heinonen E (2000). The effects of a specific alpha(2)-adrenoceptor antagonist, atipamezole, on cognitive performance and brain neurochemistry in aged Fisher 344 rats. Eur. J. Pharmacol..

[102] Veyrac A, Nguyen V, Marien M, Didier A, Jourdan F (2007). Noradrenergic control of odor recognition in a nonassociative olfactory learning task in the mouse. Learn. Mem..

[103] Chopin P, Colpaert FC, Marien M (2002). Effects of acute and subchronic administration of dexefaroxan, an alpha(2)-adrenoceptor antagonist, on memory performance in young adult and aged rodents. J. Pharmacol. Exp. Ther..

[104] Camacho F, Smith CP, Vargas HM, Winslow JT (1996). Alpha 2-adrenoceptor antagonists potentiate acetylcholinesterase inhibitor effects on passive avoidance learning in the rat. Psychopharmacol. (Berl.).

[105] Haddjeri N, Blier P, de Montigny C (1997). Effects of long-term treatment with the alpha 2-adrenoceptor antagonist mirtazapine on 5-HT neurotransmission. Naunyn Schmiedebergs Arch. Pharmacol..

[106] Kashiwagi A (1986). New alpha 2-adrenergic blocker (DG-5128) improves insulin secretion and in vivo glucose disposal in NIDDM patients. Diabetes.

[107] Abdel-Zaher AO, Ahmed IT, El-Koussi AD (2001). The potential antidiabetic activity of some alpha-2 adrenoceptor antagonists. Pharmacol. Res..

[108] Fagerholm V, Haaparanta M, Scheinin M (2011). α2-adrenoceptor regulation of blood glucose homeostasis. Basic. Clin. Pharmacol. Toxicol..

[109] Chopin P, Colpaert FC, Marien M (1999). Effects of alpha-2 adrenoceptor agonists and antagonists on circling behavior in rats with unilateral 6-hydroxydopamine lesions of the nigrostriatal pathway. J. Pharmacol. Exp. Ther..

[110] Lahousse SA, Stopa EG, Mulberg AE, de la Monte SM (2003). Reduced expression of the cystic fibrosis transmembrane conductance regulator gene in the hypothalamus of patients with Alzheimer’s disease. J. Alzheimers Dis..

[111] Uramoto H, Okada T, Okada Y (2012). Protective role of cardiac CFTR activation upon early reperfusion against myocardial infarction. Cell Physiol. Biochem..

[112] Guo Y, Su M, McNutt MA, Gu J (2009). Expression and distribution of cystic fibrosis transmembrane conductance regulator in neurons of the human brain. J. Histochem. Cytochem..

[113] Tsabari R (2016). CFTR potentiator therapy ameliorates impaired insulin secretion in CF patients with a gating mutation. J. Cyst. Fibros..

[114] Gong X (2012). Down-regulation of IGF-1/IGF-1R in hippocampus of rats with vascular dementia. Neurosci. Lett..

[115] Wang J (2013). Insulin-like growth factor-1 secreted by brain microvascular endothelial cells attenuates neuron injury upon ischemia. FEBS J..

[116] Chang HC, Yang YR, Wang PS, Kuo CH, Wang RY (2013). The neuroprotective effects of intramuscular insulin-like growth factor-I treatment in brain ischemic rats. PLoS One.

[117] Gao S (2014). MicroRNA-133a regulates insulin-like growth factor-1 receptor expression and vascular smooth muscle cell proliferation in murine atherosclerosis. Atherosclerosis.

[118] Higashi Y (2016). Insulin-Like Growth Factor-1 Receptor Deficiency in Macrophages Accelerates Atherosclerosis and Induces an Unstable Plaque Phenotype in Apolipoprotein E-Deficient Mice. Circulation.

[119] Higashi Y (2014). Interaction between insulin-like growth factor-1 and atherosclerosis and vascular aging. Front. Horm. Res..

[120] Cheng J (2008). Insulin-like growth factor-1 receptor polymorphism and ischemic stroke: a case-control study in Chinese population. Acta Neurol. Scand..

[121] Clemmons DR (2007). Modifying IGF1 activity: an approach to treat endocrine disorders, atherosclerosis and cancer. Nat. Rev. Drug. Discov..

[122] Cheng PW (2014). Tseng. Involvement of two distinct signalling pathways in IGF-1-mediated central control of hypotensive effects in normotensive and hypertensive rats. Acta Physiol. (Oxf)..

[123] Raber J (2008). AR, apoE, and cognitive function. Horm. Behav..

[124] Pike CJ, Carroll JC, Rosario ER, Barron AM (2009). Protective actions of sex steroid hormones in Alzheimer’s disease. Front. Neuroendocrinol..

[125] Pal M, Gupta S (2016). Testosterone supplementation improves glucose homeostasis despite increasing hepatic insulin resistance in male mouse model of type 2 diabetes mellitus. Nutr. Diabetes..

[126] Uchida M (2009). Dose-dependent effects of androgens on outcome after focal cerebral ischemia in adult male mice. J. Cereb. Blood Flow. Metab..

[127] Zhang W (2014). Effects of androgens on early post-ischemic neurogenesis in mice. Transl. Stroke Res..

[128] Cheng J (2009). Age-dependent effects of testosterone in experimental stroke. J. Cereb. Blood Flow. Metab..

[129] Huang CK (2014). New therapy via targeting androgen receptor in monocytes/macrophages to battle atherosclerosis. Hypertension.

[130] Fagman JB (2015). The androgen receptor confers protection against diet-induced atherosclerosis, obesity, and dyslipidemia in female mice. FASEB J..

[131] Chignalia AZ (2012). Testosterone induces vascular smooth muscle cell migration by NADPH oxidase and c-Src-dependent pathways. Hypertension.

[132] Chang C, Yeh S, Lee SO, Chang TM (2013). Androgen receptor (AR) pathophysiological roles in androgen-related diseases in skin, bone/muscle, metabolic syndrome and neuron/immune systems: lessons learned from mice lacking AR in specific cells. Nucl. Recept. Signal..

[133] Navarro G (2016). Extranuclear Actions of the Androgen Receptor Enhance Glucose-Stimulated Insulin Secretion in the Male. Cell Metab..

[134] Yoshida S (2013). Androgen receptor promotes sex-independent angiogenesis in response to ischemia and is required for activation of vascular endothelial growth factor receptor signaling. Circulation.

[135] Venna VR, Benashski SE, Chauhan A, McCullough LD (2015). Inhibition of glycogen synthase kinase-3β enhances cognitive recovery after stroke: the role of TAK1. Learn. Mem..

[136] Thareja S, Aggarwal S, Bhardwaj TR, Kumar M (2012). Protein tyrosine phosphatase 1B inhibitors: a molecular level legitimate approach for the management of diabetes mellitus. Med. Res. Rev..

[137] Hassid A, Huang S, Yao J (1999). Role of PTP-1B in aortic smooth muscle cell motility and tyrosine phosphorylation of focal adhesion proteins. Am. J. Physiol..

[138] Bruder-Nascimento T (2015). Deletion of protein tyrosine phosphatase 1b in proopiomelanocortin neurons reduces neurogenic control of blood pressure and protects mice from leptin- and sympatho-mediated hypertension. Pharmacol. Res..

[139] Belin de CEJ (2009). Protein tyrosine phosphatase 1B, a major regulator of leptin-mediated control of cardiovascular function. Circulation.

