# Supplementary material for: Combined network pharmacology and virtual reverse pharmacology approaches for identification of potential targets to treat vascular dementia
Source: Sci Rep. 2020 Jan 14;10:257. doi: 10.1038/s41598-019-57199-9 (PMC6959222; doi:10.1038/s41598-019-57199-9)
Supplement: Supplementary file 1 — Supplementary Information [file 41598_2019_57199_MOESM1_ESM.pdf]

Supplementary Materials for the article:

Combined network pharmacology and virtual reverse pharmacology approaches for identification of potential targets to treat vascular dementia

Alexey A. Lagunin[1,2*], Sergey M. Ivanov[1,2], Tatyana A. Gloriozova[2], Pavel V. Pogodin[2], Dmitry A. Filimonov[2], Sandeep Kumar[3], Rajesh K. Goel[3*]

[1]Department of Bioinformatics, Pirogov Russian National Research Medical University, Moscow, Russia.

[2]Department of Bioinformatics, Institute of Biomedical Chemistry, Moscow, Russia.

[3] Department of Pharmaceutical Sciences and Drug Research, Punjabi University, Patiala, India.

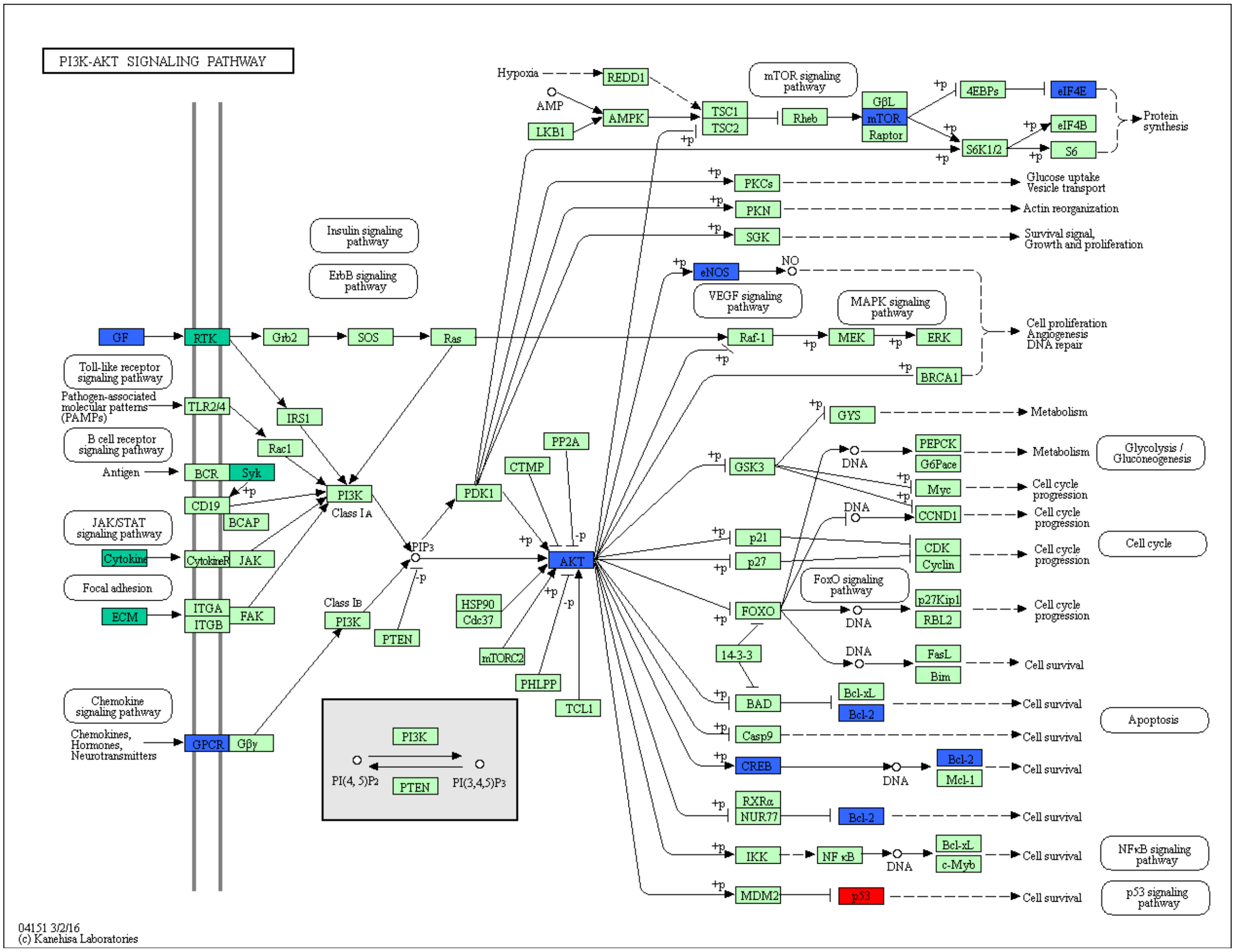

Fig. S1. PI3K-AKT signalling pathway with mapping of known genes associated with VaD. Blue color means that these genes are downregulated in VaD or activation of corresponding proteins is protective against VaD. Red color means that these genes are up-regulated in VaD or inhibition of corresponding proteins is protective against VaD. Dark green color means that these genes associated with VaD, but direction of their changes is unknown. We acknowledge Kanehisa Laboratories for permission of use the image of PI3K-AKT signalling pathway from KEGG database [Kanehisa, M. and Goto, S. KEGG: Kyoto Encyclopedia of Genes and Genomes. Nucleic Acids Res. 2000, 28, 27-30].

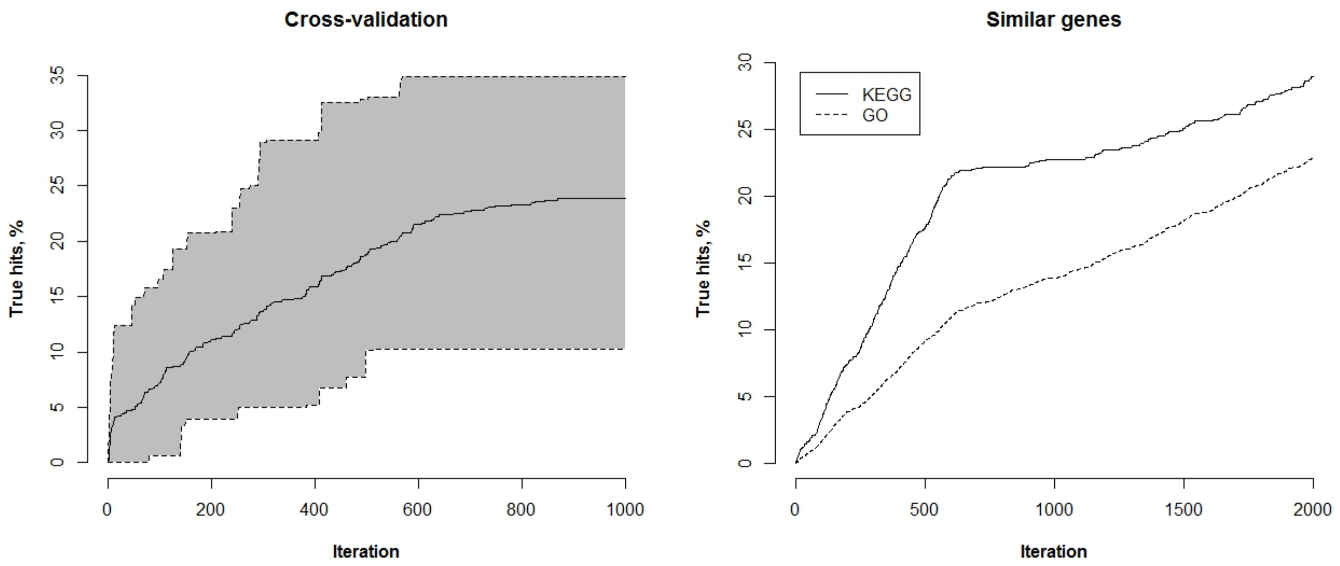

Fig. S2. Relationship between the iteration of the algorithm and percentage of VaD-related genes (left) or functionally similar genes (right) included in the growing module (true hits, %). The left figure shows the average curve and 95% confidence interval calculated by multiple repeats of the 5-fold cross-validation procedure. The right figure shows curves for functionally similar genes from KEGG pathways and Gene Ontology (GO) biological processes which are enriched by VaD-related genes.

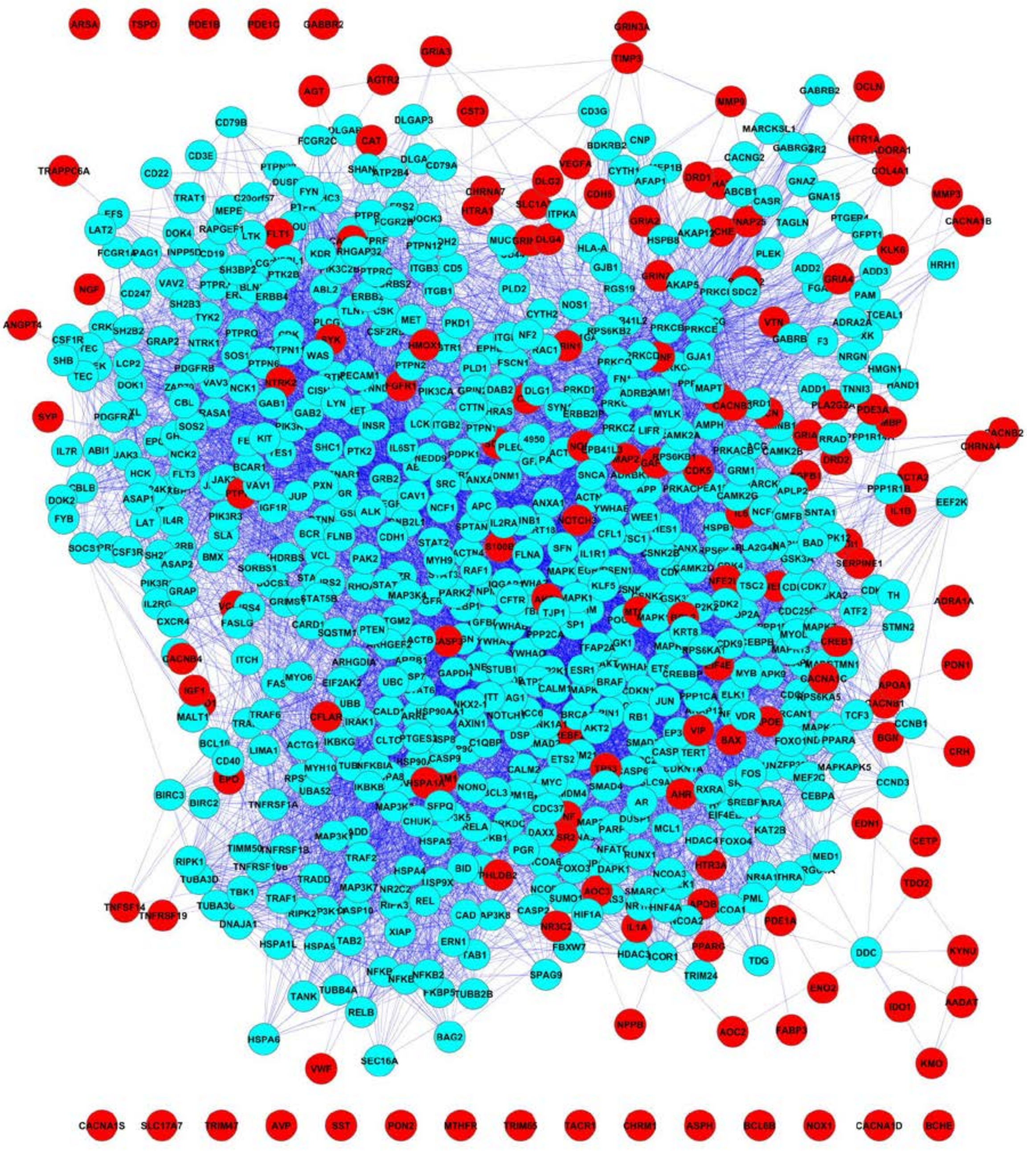

Fig. S3. VaD-related network module. Red nodes – known VaD-related proteins. Blue nodes – selected targets by DIAMOnD algorithm.

Table S1. One hundred one genes correlated with vascular dementia.

Name	Gene	Subtype of VD	Species	Alteration	Relationship	PubMedID
**72 kDa type IV collagenase**	MMP2	Vascular dementia	r	increased expression	correlates with	16385583
**Actin, aortic smooth muscle**	ACTA2	CADASIL	h	decreased expression	correlates with	18176893
**Angiopoietin-4**	ANGPT4	Mixed	h	polymorphism	correlates with	20596041
**Angiotensinogen**	AGT	Vascular dementia	h	polymorphism	correlates with	16603315; 19550366; 16603315; 16603315; 19550366
**Apolipoprotein A-I**	APOA1	Vascular dementia	h	decreased expression; increased expression	correlates with	14531942; 10828089
**Apolipoprotein B-100**	APOB	Vascular dementia	h	increased expression	correlates with	14531942
**Apolipoprotein E**	APOE	Vascular dementia	h	polymorphism; increased expression	correlates with	7841371; 20375505; 10828089
**Apoptosis regulator BAX**	BAX	Multi-infarct dementia	r	increased expression	correlates with	18938189
**Apoptosis regulator Bcl-2**	BCL2	Multi-infarct dementia	r	decreased expression	correlates with	18938189
**Arylsulfatase A**	ARSA	Vascular dementia	h	mutation	correlates with	9261837
**Aspartyl/asparaginyl beta-hydroxylase**	ASPH	CADASIL	h	decreased expression	correlates with	21504125
**B-cell CLL/lymphoma 6 member B protein**	BCL6B	Subcortical vascular dementia	r	expression	correlates with	27458816
**BDNF/NT-3 growth factors receptor**	NTRK2	Vascular dementia	r, m	decreased expression	correlates with	25585610; 25126168
**Beta-nerve growth factor**	NGF	Vascular dementia	h, r	decreased expression	correlates with	17361527; 16364207; 9868540
**Biglycan**	BGN	CADASIL	h	increased expression	correlates with	25578324
**Cadherin-6**	CDH6	CADASIL	h	decreased expression	correlates with	25870235
**CASP8 and FADD-like apoptosis regulator**	CFLAR	CADASIL	h	decreased expression	correlates with	19419383; 11925448
**Caspase-3**	CASP3	CADASIL	h	increased phosphorylation	correlates with	25251607; 24455679
**Catalase**	CAT	Vascular dementia	r	decreased expression	correlates with	25966684; 24597602
**Cellular tumor antigen p53**	TP53	Vascular dementia	r	increased expression	correlates with	18083315
**Cholesteryl ester transfer protein**	CETP	Vascular dementia	h	polymorphism	correlates with	19184337
**Choline O-acetyltransferase**	CHAT	Vascular dementia	h	increased activity	correlates with	19776575

**Corticoliberin**	CRH	Vascular dementia	h	decreased expression	correlates with	8547442
**C-reactive protein**	CRP	Vascular dementia	h	increased expression	correlates with	18841009
**Cyclic AMP-responsive element-binding protein 1**	CREB1	Vascular dementia	r	decreased expression	correlates with	25481359; 26094797
**Cyclin-dependent-like kinase 5**	CDK5	Vascular dementia	r	increased activity	correlates with	22621232; 27118553
**Cystatin-C**	CST3	Vascular dementia	h	polymorphism	correlates with	14672279
**Cysteine-rich secretory protein LCCL domain-containing 2**	CRISPLD 2	Subcortical vascular dementia	r	expression	correlates with	27458816
**Decorin**	DCN	CADASIL	h	increased expression	correlates with	25578324
**Disks large homolog 2**	DLG2	Vascular dementia	r	decreased expression	correlates with	26592482
**Disks large homolog 4**	DLG4	Vascular dementia	r, m	decreased expression	correlates with	26179091; 25172625; 23535468
**Endothelin-1**	EDN1	Vascular dementia	r	increased expression	correlates with	25821478; 26427105
**Erythropoietin**	EPO	Vascular dementia	r	expression	correlates with	17037738
**Estrogen receptor beta**	ESR2	Vascular dementia	h	polymorphism	correlates with	19473078; 22183267
**Eukaryotic translation initiation f...**	EIF4E	Vascular dementia	r	decreased expression	correlates with	23053837
**Excitatory amino acid transporter 2**	SLC1A2	Vascular dementia	h	increased expression	correlates with	25497727
**Fatty acid-binding protein, heart**	FABP3	Vascular dementia	h	increased expression	correlates with	23254629; 22163231
**Fibroblast growth factor receptor 1**	FGFR1	Vascular dementia	r	expression	correlates with	22500404
**Gamma-enolase**	ENO2	Multi-infarct dementia	h	increased expression	correlates with	26770594; 7796631
**Glutamate receptor 2**	GRIA2	Subcortical vascular dementia	h	increased expression	correlates with	21419184
**Glutamate receptor ionotropic, NMDA 1**	GRIN1	Vascular dementia	r	decreased expression	correlates with	25261450; 25541037; 19953343
**Glutamate receptor ionotropic, NMDA 2A**	GRIN2A	Vascular dementia	h, r, m	decreased expression	correlates with	25261450; 25420607; 25773786
**Glutamate receptor ionotropic, NMDA 2B**	GRIN2B	Vascular dementia	r	decreased expression	correlates with	27086971; 25261450; 19097500
**Glutathione S-transferase omega-1**	GSTO1	Vascular dementia	h	polymorphism	correlates with	15623683; 17717316
**Heat shock 70 kDa protein 1A**	HSPA1A	Vascular dementia	h	polymorphism	correlates with	15832029
**Heme oxygenase 1**	HMOX1	Vascular dementia	r, m	decreased expression	correlates with	18174021; 26966782

**Insulin-like growth factor I**	IGF1	Vascular dementia	r	polymorphism; decreased expression	correlates with	16983186; 22342912
**Intercellular adhesion molecule 1**	ICAM1	Vascular dementia	h	polymorphism	correlates with	12095649
**Interleukin-1 alpha**	IL1A	Vascular dementia	h	polymorphism	correlates with	17622713
**Interleukin-1 beta**	IL1B	Vascular dementia	h	polymorphism	correlates with	16226351
**Interleukin-6**	IL6	Vascular dementia	h	polymorphism	correlates with	22015309; 21252539
**Kallikrein-6**	KLK6	Vascular dementia	h	increased expression	correlates with	20846516
**Matrix metalloproteinase-9**	MMP9	Vascular dementia	h	polymorphism; increased expression	correlates with	17077200; 25024308
**Membrane primary amine oxidase**	AOC3	Multi-infarct dementia	h	increased expression	correlates with	11872247
**Metalloproteinase inhibitor 3**	TIMP3	CADASIL	m	increased expression	correlates with	26648042
**Microtubule-associated protein 2**	MAP2	Vascular dementia	r	decreased expression	correlates with	26179091; 24339978; 23022689
**Muscarinic acetylcholine receptor M1**	CHRM1	Vascular dementia	r	decreased expression	correlates with	8861111; 1446228
**Myelin basic protein**	MBP	Vascular dementia	r, m	decreased expression	correlates with	26597908; 25898017; 22943141
**NADPH oxidase 1**	NOX1	Vascular dementia	r	increased expression	correlates with	25808015; 24294978
**Natriuretic peptides B**	NPPB	Subcortical vascular dementia	h	increased expression	correlates with	19424098
**Neurofilament light polypeptide**	NEFL	Vascular dementia	h	increased expression	correlates with	15717022; 14499942
**Neuromodulin**	GAP43	Vascular dementia	r, m	decreased expression	correlates with	25737708; 24957473
**Neuronal acetylcholine receptor subunit alpha-4**	CHRNA4	Vascular dementia	h	decreased expression	correlates with	26858154
**Nitric oxide synthase, endothelial**	NOS3	Vascular dementia	h	decreased expression	correlates with	25760219; 26104027; 18839024; 20406638; 18537053
**Nuclear factor erythroid 2-related factor 2**	NFE2L2	Vascular dementia	r	decreased expression	correlates with	25808015
**Occludin**	OCLN	Vascular dementia	h	increased expression	correlates with	17635647
**Phospholipase A2, membrane associat...**	PLA2G2A	Vascular dementia	h	increased activity	correlates with	17447002
**Plasminogen activator inhibitor 1**	SERPINE1	Vascular dementia	h	increased expression	correlates with	8815562
**Pleckstrin homology-like domain fam...**	PHLDB2	Vascular dementia	h	polymorphism	correlates with	22111664

**Protein fantom**	RPGRIP1L	Vascular dementia	h	polymorphism	correlates with	22425971
**Protein S100-B**	S100B	Vascular dementia	h	increased expression	correlates with	23356138
**Rab GDP dissociation inhibitor alpha**	GDI1	Vascular dementia	h	decreased expression	correlates with	25497727
**RAC-alpha serine/threonine-protein kinase**	AKT1	Vascular dementia	h	decreased phosphorylation	correlates with	26240057; 26523278; 26208699
**Receptor-type tyrosine-protein phosphatase beta**	PTPRB	Vascular dementia	r	expression	correlates with	27458816
**Reticulophagy receptor FAM134B**	FAM134B	Vascular dementia	h	polymorphism	correlates with	21127458
**Retina-specific copper amine oxidase**	AOC2	Vascular dementia	h	increased activity	correlates with	18845121
**Serine/threonine-protein kinase mTO...**	MTOR	Vascular dementia	r	decreased expression	correlates with	23053837
**Serum paraoxonase/arylesterase 1**	PON1	Vascular dementia	h	decreased expression	correlates with	12480737; 24965284; 12480756
**Serum paraoxonase/arylesterase 2**	PON2	Vascular dementia	h	polymorphism	correlates with	11803456
**Somatostatin**	SST	Vascular dementia	r	decreased expression	correlates with	18925713
**Sterol regulatory element-binding p...**	SREBF2	Vascular dementia	h	polymorphism	correlates with	16082694
**Stromelysin-1**	MMP3	Vascular dementia	h	polymorphism	correlates with	17077200
**Superoxide dismutase [Cu-Zn]**	SOD1	CADASIL	h	decreased expression	correlates with	23036509
**Synaptophysin**	SYP	Vascular dementia	h	decreased expression	correlates with	25559750; 25737708
**Synaptosomal-associated protein 25**	SNAP25	Vascular dementia	h	expression	correlates with	25559750; 25497727
**Trafficking protein particle complex subunit 6A**	TRAPPC6A	Vascular dementia	r	increased expression	correlates with	27458816
**Transforming growth factor beta-1**	TGFB1	Vascular dementia	h	polymorphism	correlates with	16990569
**Tripartite motif-containing protein 47**	TRIM47	Subcortical vascular dementia	h	polymorphism	correlates with	22735669
**Tripartite motif-containing protein 65**	TRIM65	Subcortical vascular dementia	h	polymorphism	correlates with	22735669
**Tumor necrosis factor**	TNF	Multi-infarct dementia	h	increased expression	correlates with	12505423
**Tumor necrosis factor ligand superf**	TNFSF14	Vascular dementia	h	polymorphism	correlates with	18320356
**Tumor necrosis factor receptor superfamily member 19**	TNFRSF19	Vascular dementia	h	polymorphism	correlates with	21127458
**Tyrosine-protein kinase SYK**	SYK	Vascular dementia	h	polymorphism	correlates with	23480133

**Vascular cell adhesion protein 1**	VCAM1	Vascular dementia	h	increased expression	correlates with	18597785
**Vascular endothelial growth factor**	VEGFA	Vascular dementia	h	increased expression	correlates with	11804709
**Vascular endothelial growth factor ...**	FLT1	Vascular dementia	r	expression	correlates with	22500404
**Vasopressin-neurophysin 2-copeptin**	AVP	Vascular dementia	r	decreased expression	correlates with	18925713
**Vesicular glutamate transporter 1**	SLC17A7	Vascular dementia	h	decreased expression; increased expression	correlates with	21079182
**VIP peptides**	VIP	Vascular dementia	h	increased expression	correlates with	10864607
**Vitronectin**	VTN	CADASIL	m	increased expression	correlates with	26648042
**von Willebrand factor**	VWF	Vascular dementia	h	increased expression	correlates with	8815562; 22639698

Species, h – human, r – rat, m – mouse.

Table S2. General GO biological processes associated with VaD according to DAVID.

Process	Description	PubMed ID
**Angiogenesis**	Promotion of angiogenesis may have therapeutic effects on VaD.	24589546
**Atherosclerotic processes (i.e., lipid/cholesterol transport, smooth muscle cell migration, proliferation, apoptosis, and foam cell differentiation)**	Atherosclerosis is a risk factor for VaD because it causes narrowing of cerebral arteries and enhances stroke risk.	21557721; 27284205
**Autophagy**	Autophagy activation aggravates neuronal injury.	25221581
**Cell-cell junction and cell-matrix adhesion**	Cell adhesion and cell junctions have roles in many cellular processes associated with VaD.	23865428; 17635647; 25870235
**Cognition, learning, memory, behaviour**	These processes are disrupted in VaD.	26595643
**Glucose homeostasis/response to insulin**	Insulin resistance impacts VaD through multiple mechanisms.	27303627
**Inflammatory response**	Neuroinflammation plays an important role in VaD. Inflammation also has a key role in atherosclerosis.	26725994; 26714236
**Neurogenesis and gliogenesis**	Neurogenesis improves cognitive function in VaD.	26934837; 25555543
**Neuron apoptosis**	Cell death significantly contributes to VaD pathogenesis.	24660032; 25541037
**Regulation of haemostasis**	There is an established relationship between haemostasis and dementia.	20167665
**Regulation of transcription and translation**	Decreased protein expression in VaD.	25015703
**Regulation of vascular tone and blood pressure**	Increased cerebral vasoconstriction in VaD as well as hypertension is a known risk factor.	24003901
**Response to oxidative stress**	One of the key pathogenic mechanisms of VaD.	27662637; 22519891
**Synaptic signalling**	Reduced glutamatergic and cholinergic transmission in VaD.	27539743; 22285808; 22191561
**Kynurenine metabolism**	Metabolites of kynurenine play an important role in the pathogenesis of VaD.	25248805

Table S3. KEGG pathways related to VaD, which were revealed with DAVID web service during gene set enrichment analysis.

Pathway	N	Adjusted p-value	Description
**AMPK signalling pathway**	6	9.8E-2	Regulation of protein synthesis, autophagy and gluconeogenesis.
**Apoptosis**	8	4.3E-4	Regulation of apoptosis.
**cAMP signalling pathway**	10	1.2E-2	Regulation of the AKT pathway, excitability and cell survival/death in hippocampal neurons as well as regulation of intracellular calcium.
**Cholinergic synapse**	7	2.8E-2	Cell survival and synaptic plasticity.
**Cytokine-cytokine receptor interaction**	10	2.8E-2	Regulation of many VaD-related processes.
**Oestrogen signalling pathway**	8	5.2E-3	Regulation of apoptosis and cell adhesion as well as membrane and cytoplasmic signalling cascades.
**Focal adhesion**	8	9.2E-2	Regulation of the cytoskeleton and focal adhesion.
**Glutamatergic synapse**	7	2.8E-2	Long-term potentiation.
**HIF-1 signalling pathway**	15	4.0E-9	Regulation of translation, angiogenesis, vascular tone and apoptosis.
**Leukocyte transendothelial migration**	6	9.4E-2	Transendothelial migration of leukocytes in inflammation.
**MAPK signalling pathway**	12	7.6E-3	Regulation of multiple cell functions, including cell survival and apoptosis.
**mTOR signalling pathway**	5	4.4E-2	Regulation of protein synthesis, cell survival, autophagy, lipid metabolism and cytoskeleton.
**Neuroactive ligand-receptor interaction**	11	2.9E-2	Regulation of many VaD-related processes.
**Neurotrophin signalling pathway**	7	3.5E-2	Regulation of cell survival and apoptosis as well as the cytoskeleton, retrograde transport, axonal growth, guidance, patterning and synapse formation. These processes contribute to neurogenesis.
**NF-kappa B signalling pathway**	8	2.7E-3	Regulation of cell survival and inflammation.
**p53 signalling pathway**	5	6.2E-2	Regulation of apoptosis.
**PI3K-Akt signalling pathway**	20	1.0E-5	Regulation of translation, angiogenesis and cell survival.
**Rap1 signalling pathway**	10	1.7E-2	Regulation of cell adhesion, cell junctions, the cytoskeleton, cell survival, cell migration and angiogenesis.
**Ras signalling pathway**	11	9.4E-3	Regulation of cell survival, apoptosis, cell-cell junctions, cytoskeletal remodelling and endocytosis.
**Sphingolipid signalling pathway**	6	9.4E-2	Regulation of vasodilatation and apoptosis. No sphingolipid receptors among VaD-related genes.
**TNF signalling pathway**	12	8.6E-6	Regulation of apoptosis and survival, cell adhesion, extracellular matrix remodelling, vascular effects and synthesis of inflammatory cytokines.
**Tryptophan metabolism**	7	3.7E-4	Metabolites of kynurenine play important roles in the pathogenesis of VaD.

N – number of VaD-related genes in the appropriate KEGG pathway. Adjusted p-value – p-value corrected to control for testing multiple hypotheses and represents the minimum false discovery rate for which the correlation will be regarded as significant. The adjusted p-values were calculated using the Benjamini-Hochberg procedure.

Table S4. Diseases associated with VaD-related genes revealed by disease enrichment in the PROTEOME database.

Disease	N of genes	p-value	Disease	N of genes	p-value
**Parkinson Disease**	56	0	Pancreatic Neoplasms	43	$4.22E^{-24}$
**Alzheimer Disease**	86	0	Polycystic Ovary Syndrome	26	$1.01E^{-23}$
**Brain Ischemia**	30	$1.12E^{-44}$	**Hypercholesterolemia**	21	$2.19E^{-23}$
**Obesity**	52	$6.14E^{-37}$	Multiple Sclerosis, Relapsing-Remitting	22	$2.33E^{-23}$
**Multiple Sclerosis**	50	$5.91E^{-36}$	Pulmonary Disease, Chronic Obstructive	28	$2.46E^{-23}$
**Arteriosclerosis**	35	$8.06E^{-35}$	Huntington Disease	35	$3.20E^{-23}$
**Bipolar Disorder**	41	$2.29E^{-34}$	Glioma	34	$4.94E^{-23}$
**Prostatic Neoplasms**	64	$5.60E^{-34}$	Ovarian Neoplasms	51	$8.95E^{-23}$
**Amyotrophic Lateral Sclerosis**	36	$2.45E^{-33}$	Vitiligo	19	$1.55E^{-22}$
**Dementia, Vascular**	17	$3.58E^{-33}$	**Carotid Artery Diseases**	16	$4.27E^{-22}$
**Coronary Disease**	29	$5.47E^{-32}$	Myocardial Infarction	24	$1.20E^{-21}$
**Stroke**	26	$6.12E^{-32}$	Glaucoma, Open-Angle	16	$1.71E^{-21}$
**Hypertension**	40	$1.33E^{-30}$	Carcinoma, Small Cell	23	$4.56E^{-21}$
**Schizophrenia**	52	$2.36E^{-30}$	HIV Infections	32	$5.78E^{-21}$
**Coronary Artery Disease**	37	$3.21E^{-30}$	Glioblastoma	42	$8.00E^{-21}$
**Arthritis, Rheumatoid**	49	$5.55E^{-30}$	Anemia, Sickle Cell	23	$8.07E^{-21}$
**Diabetes Mellitus, Type 2**	45	$7.52E^{-30}$	Asthma	30	$1.22E^{-20}$
**Lung Neoplasms**	66	$2.23E^{-29}$	Breast Neoplasms	73	$2.12E^{-20}$
**Depressive Disorder, Major**	30	$1.48E^{-27}$	Diabetic Nephropathies	21	$4.77E^{-20}$
**Atherosclerosis**	31	$1.12E^{-26}$	Inflammatory Bowel Diseases	20	$1.42E^{-19}$
**Lupus Erythematosus, Systemic**	35	$2.38E^{-25}$	**Hyperlipoproteinemia Type II**	13	$1.78E^{-19}$
**Diabetes Mellitus, Type 1**	32	$6.25E^{-25}$	Carcinoma, Non-Small-Cell Lung	64	$1.85E^{-19}$
**Diabetes Mellitus**	23	$1.32E^{-24}$	Erectile Dysfunction	14	$5.28E^{-19}$
**Psoriasis**	42	$1.94E^{-24}$	Liver Neoplasms	25	$5.79E^{-19}$
**Migraine Disorders**	27	$3.42E^{-24}$	Colonic Neoplasms	43	$6.27E^{-19}$

Bold means that these disorder are known risk factors for VaD.

Table S5. Drugs and their targets that are used for the treatment of VaD.

Target	Action	Gene	Therapeutic effects	PubMed ID	Drugs
**Adenosine receptor A1**	Antagonist	ADORA1	Alzheimer's disease treatment; Antihypertensive; Antiparkinsonian; Cognition disorders treatment; Nootropic; Antihypercholesterolemic	14982979; 15351792; 11164065; 21185259	Aminophylline
**Adenosine receptor A3**	Antagonist	ADORA3	Antiischemic, cerebral; Stroke treatment	10355598; 12570761	Aminophylline
**cGMP-inhibited 3',5'-cyclic phosphodiesterase A**	Inhibitor	PDE3A	Antidiabetic symptomatic*; Antithrombotic*; Atherosclerosis treatment*; Neuroprotector*	Prous, 1995	Aminophylline
**Glutamate receptor ionotropic, NMDA 2A**	Antagonist	GRIN2A**	Neuroprotector*	14987468	Glycine, Memantine
**5-hydroxytryptamine receptor 3A**	Antagonist	HTR3A	Cognition disorders treatment*, Age-associated memory impairment treatment*, Nootropic*, Stroke treatment*	2140610; 12130738	Memantine
**Alpha-7 nicotinic cholinergic receptor subunit**	Antagonist	CHRNA7	Alzheimer's disease treatment	19721446	Memantine
**D(2) dopamine receptor**	Agonist	DRD2	Antihypertensive	1356782; 9055350	Memantine
**Glutamate receptor ionotropic, NMDA 2B**	Antagonist	GRIN2B**	Neuroprotector*; Stroke treatment	14987468	Memantine
**Glutamate receptor ionotropic, NMDA 3A**	Antagonist	GRIN3A	Alzheimer's disease treatment	16377242; 14530799	Memantine
**Alpha-1A adrenergic receptor**	Antagonist	ADRA1A**	Alzheimer's disease treatment*; Antihypertensive*; Nootropic*	Health Technology Assessment 2001, 5 (1); Prous, 1995	Nicergoline
**Mineralocorticoid receptor**	Antagonist	NR3C2	Antihypertensive	Pharmaprojects V5, 2007	Nimodipine
**Voltage-dependent L-type calcium channel subunit alpha-1C**	Inhibitor	CACNA1C	Antihypertensive*	12382411	Nimodipine
**Voltage-dependent L-type calcium channel subunit alpha-1D**	Inhibitor	CACNA1D	Antihypertensive*	12382411	Nimodipine
**Voltage-dependent L-type calcium channel subunit alpha-1F**	Inhibitor	CACNA1F	Antihypertensive*	12382411	Nimodipine
**Voltage-dependent L-type calcium channel subunit alpha-1S**	Inhibitor	CACNA1S	Antihypertensive*	12382411	Nimodipine
**Voltage-dependent L-type**	Inhibitor	CACNB1	Antihypertensive*	12382411	Nimodipine

**calcium channel subunit beta-1**					
**Voltage-dependent L-type calcium channel subunit beta-2**	Inhibitor	CACNB2	Antihypertensive*	12382411	Nimodipine
**Voltage-dependent L-type calcium channel subunit beta-3**	Inhibitor	CACNB3	Antihypertensive*	12382411	Nimodipine
**Voltage-dependent L-type calcium channel subunit beta-4**	Inhibitor	CACNB4	Antihypertensive*	12382411	Nimodipine
**Glutamate receptor ionotropic AMPA**	Stimulator	GRIA1; GRIA2**; GRIA3; GRIA4	Neuroprotector; Nootropic	Prous, 1995	Piracetam
**Voltage-dependent N-type calcium channel subunit alpha-1B**	Inhibitor	CACNA1B	Antiischemic, cerebral	1453701	Piracetam
**D(1A) dopamine receptor**	Agonist	DRD1	Antihypertensive; Antiparkinsonian; Nootropic	7715795	Vinpocetine
**Phosphodiesterase 1**	Inhibitor	PDE1A; PDE1B; PDE1C	Antihypertensive*; Antiparkinsonian*; Antithrombotic*; Nootropic*; Restenosis treatment*	12177649	Vinpocetine
**Translocator protein**	Binding	TSPO	Neuroprotector	22874716	Vinpocetine

* - this relationship is known for common formulation of targets, e.g. “Alpha-1 adrenergic receptor” instead of “Alpha-1A adrenergic receptor”, but other supporting information (e.g. polymorphisms, association with another neurodegenerative disease, etc.) for this sub-class of targets is available; ** - these genes were in the list of 122 genes associated with VaD.

Table S6. Fourteen KEGG pathways significantly overlapped with 600 predicted VaD-related proteins.

**Pathway**	**No of proteins**	**Mean "600-rank" value**	**Overlap, %**	**Pubmed ID**
Adherens junction	40	307.7	55.6	19318941; 18343578; 21705496
Chemokine signaling pathway	63	394.8	33.7	23482064; 23168453
ErbB signaling pathway	60	397.9	68.2	26442853; 25985800; 26093380; 15694257
Estrogen signaling pathway	38	368.8	38	19473078; 22183267; 20616674; 24772998
Fc gamma R-mediated phagocytosis	47	416.5	50.5	25430817; 27256292; 25135788
FoxO signaling pathway	53	325.9	39.6	27317635; 26191159; 25722793; 27651777; 25609639
Gap junction	29	369	33.0	22960118; 22238107
Insulin signaling pathway	59	373.3	42.1	22956272; 23627981; 24931034
Jak-STAT signaling pathway	49	381.2	31.0	10756075; 21385378; 14730712; 17880360
Longevity regulating pathway - multiple species	22	382.4	34.4	26442674
Long-term potentiation	29	366	43.3	28664509; 28289242
Prolactin signaling pathway	42	365.7	58.3	17317019
Thyroid hormone signaling pathway	52	327.4	44.1	24577884; 14761671; 16966610
VEGF signaling pathway	32	404.4	52.5	24589546

Table S7. PASS mechanisms of action related to known targets associated with VaD, the number of active (Number) compounds in the PASS training set and the accuracy of their prediction (AUC) calculated by leave-one-out cross-validation (LOO CV).

No	Mechanism of action	Number	AUC
1	5 Hydroxytryptamine 1A agonist	1205	0.991
2	Acetylcholinesterase inhibitor	3107	0.977
3	Adrenaline uptake inhibitor	3088	0.989
4	Alpha 1a adrenoreceptor antagonist	2912	0.987
5	Angiotensin AT2 receptor agonist	7	0.989
6	Apolipoprotein B-100 inhibitor	92	0.999
7	Butyrylcholinesterase inhibitor	1588	0.987
8	Caspase 3 inhibitor	1077	0.976
9	Choline acetyltransferase inhibitor	7	0.743
10	Endothelin A receptor antagonist	2121	0.998
11	Endothelin B receptor antagonist	1168	0.997
12	Endothelin converting enzyme 1 inhibitor	179	0.995
13	Endothelin receptor antagonist	2765	0.996
14	Endothelin-converting enzyme 1 inhibitor	319	0.993
15	Excitatory amino acid transporter 2 inhibitor	49	1.000
16	GABA B receptor agonist	23	0.984
17	Glutamate (mGluR2) antagonist	279	1.000
18	Kallikrein 6 inhibitor	4	1.000
19	Metalloproteinase-3 inhibitor	1017	0.992
20	Metalloproteinase-9 inhibitor	2291	0.989
21	NADPH oxidase 1 inhibitor	61	0.997
22	Peroxisome proliferator-activated receptor gamma agonist	2531	0.989
23	Phospholipase A2 IIa inhibitor	256	0.997
24	Plasminogen activator inhibitor antagonist	308	0.994
25	Substance P antagonist	1648	0.993
26	Tumour necrosis factor antagonist	904	0.937
27	VCAM-1 antagonist	173	0.98
28	Vascular endothelial growth factor antagonist	12725	0.964
29	Vitronectin receptor antagonist	1196	0.998
30	p53 inhibitor	23	0.913
31	Indoleamine-pyrrole 2,3-dioxygenase inhibitor	216	0.992
32	Kynureninase inhibitor	43	0.991
33	Kynurenine 3 monooxygenase inhibitor	90	0.995
34	Kynurenine aminotransferase II inhibitor	34	0.965

Table S8. Prediction of mechanisms of action and pharmacological effects related to the treatment of VaD for phytomolecules of Physostigma venenosum.

PubChem ID, name	Pa	Pi	Activity	Structure
65719, eseridine	0.987	0.001	Acetylcholinesterase inhibitor	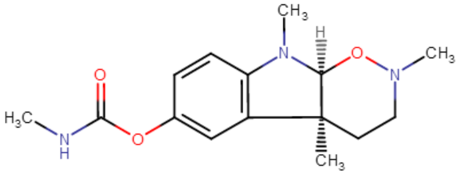
	0.982	0.002	Cholinergic	
	0.932	0.001	Butyrylcholinesterase inhibitor	
	0.899	0.004	Cognition disorders treatment	
	0.620	0.008	Nootropic	
5983, physostigmine	0.986	0.001	Acetylcholinesterase inhibitor	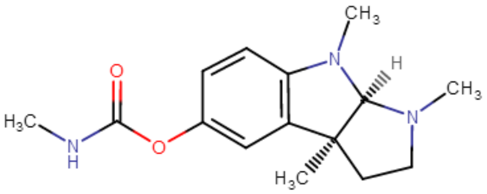
	0.982	0.002	Cholinergic	
	0.924	0.003	Cognition disorders treatment	
	0.910	0.001	Butyrylcholinesterase inhibitor	
	0.705	0.005	Alzheimer's disease treatment	
	0.627	0.005	Psychostimulant	
	0.581	0.011	Nootropic	
442077, eseramine	0.954	0.002	Cholinergic	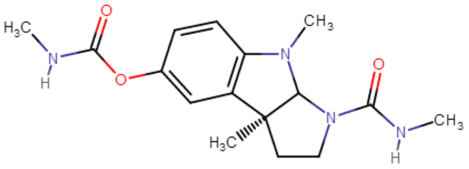
	0.928	0.001	Acetylcholinesterase inhibitor	
	0.862	0.004	Cognition disorders treatment	
	0.810	0.001	Butyrylcholinesterase inhibitor	
442113, physovenine	0.968	0.001	Acetylcholinesterase inhibitor	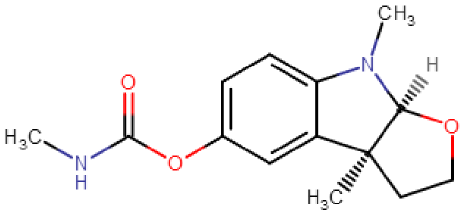
	0.846	0.003	Cholinergic	
	0.802	0.001	Butyrylcholinesterase inhibitor	
	0.651	0.007	Cognition disorders treatment	

Table S9. PASS mechanisms of action related to new targets associated with VaD, the number of active (Number) compounds in the PASS training set and the accuracy of their prediction (AUC) calculated by leave-one-out cross-validation.

No	Mechanism of action	Number	AUC
1	ALK inhibitor	1754	0.968
2	AXL receptor tyrosine kinase inhibitor	691	0.962
3	Abl kinase inhibitor	2416	0.968
4	Alpha 2a adrenoreceptor agonist	23	0.997
5	Alpha 2a adrenoreceptor antagonist	358	0.983
6	Alpha tubulin antagonist	130	0.980
7	Amyloid beta precursor protein antagonist	170	0.987
8	Androgen agonist	633	0.983
9	Androgen antagonist	1604	0.980
10	Angiotensin AT1 receptor agonist	5	0.910
11	Angiotensin AT1 receptor antagonist	4593	0.991
12	Angiotensin AT1A receptor antagonist	717	0.997
13	Baculoviral IAP repeat-containing protein 2 inhibitor	105	1.000
14	Baculoviral IAP repeat-containing protein 4 inhibitor	161	0.991
15	Bcl-xL inhibitor	220	0.977
16	Beta 2 adrenoreceptor antagonist	482	0.994
17	Beta tubulin antagonist	210	0.992
18	Bradykinin B2 receptor agonist	9	1.000
19	Bradykinin B2 receptor antagonist	652	0.998
20	CDC25A inhibitor	145	0.982
21	CDC25B inhibitor	279	0.988
22	CDC25C inhibitor	51	0.979
23	CF transmembrane conductance regulator agonist	12	0.837
24	CF transmembrane conductance regulator antagonist	332	0.992
25	CXC chemokine 4 receptor antagonist	173	0.990
26	Ca2+/calmodulin-dependent kinase II alpha inhibitor	93	0.871
27	Ca2+/calmodulin-dependent kinase II delta inhibitor	325	0.953
28	Calcium-sensing receptor agonist	140	0.996
29	Calcium-sensing receptor antagonist	306	0.997
30	Caspase 6 inhibitor	113	0.999
31	Caspase 7 inhibitor	352	1.000
32	Caspase 8 inhibitor	241	0.993
33	Caspase 8 stimulant	31	0.860
34	Caspase 9 inhibitor	40	0.969
35	Caspase 9 stimulant	39	0.790
36	Catenin beta inhibitor	12	0.911
37	Cyclin-dependent kinase 1 inhibitor	1357	0.967

38	Cyclin-dependent kinase 2 inhibitor	2299	0.965
39	Cyclin-dependent kinase 4 inhibitor	656	0.985
40	Cyclin-dependent kinase 6 inhibitor	64	0.972
41	Cyclin-dependent kinase 7 inhibitor	247	0.929
42	Cyclin-dependent kinase 9 inhibitor	233	0.928
43	Cytosolic phospholipase A2 inhibitor	266	0.997
44	DNA-dependent protein kinase inhibitor	733	0.991
45	Dihydroorotase inhibitor	64	0.995
46	EphB2 antagonist	25	0.913
47	Epidermal growth factor receptor kinase inhibitor	7537	0.968
48	ErbB-2 antagonist	2153	0.975
49	ErbB-4 antagonist	151	0.956
50	Erythropoietin receptor agonist	10	1.000
51	Erythropoietin receptor antagonist	4	1.000
52	Estrogen receptor alpha antagonist	1034	0.968
53	Factor XIIIa inhibitor	229	0.992
54	Factor XIIIa stimulant	3	0.998
55	Focal adhesion kinase 1 inhibitor	434	0.960
56	GABA A receptor agonist	745	0.978
57	GABA A receptor antagonist	2552	0.992
58	Glucocorticoid agonist	889	0.997
59	Glucocorticoid antagonist	1204	0.995
60	Glutamate (mGluR1) agonist	113	0.999
61	Glutamate (mGluR1) antagonist	1353	0.993
62	Glyceraldehyde-3-phosphate dehydrogenase inhibitor	18	0.989
63	Glycogen synthase kinase-3 alpha inhibitor	820	0.958
64	Glycogen synthase kinase-3 beta inhibitor	5930	0.974
65	Granulocyte macrophage colony stimulating factor agonist	12	0.979
66	Granulocyte macrophage colony stimulating factor antagonist	885	0.966
67	Growth factor receptor-bound protein 2 antagonist	136	0.997
68	HSPB1 expression inhibitor	12	0.805
69	Heat shock protein 90 beta antagonist	138	0.996
70	Hepatocyte growth factor agonist	7	0.869
71	Hepatocyte growth factor antagonist	1975	0.967
72	Histamine H1 receptor agonist	53	0.986
73	Histamine H1 receptor antagonist	1598	0.985
74	Histone acetyltransferase PCAF inhibitor	40	0.971
75	Histone acetyltransferase p300 inhibitor	14	0.903
76	Histone deacetylase 3 inhibitor	299	0.994
77	Histone deacetylase 4 inhibitor	244	0.998
78	Hypoxia inducible factor 1 alpha inhibitor	123	0.964

79	I kappa B kinase 1 inhibitor	496	0.979
80	I kappa B kinase 2 inhibitor	1868	0.985
81	Insulin antagonist	901	0.964
82	Insulin like growth factor 1 agonist	8	0.816
83	Insulin like growth factor 1 antagonist	2931	0.977
84	Interleukin 2 agonist	17	0.906
85	Interleukin 2 antagonist	235	0.953
86	Janus tyrosine kinase 2 inhibitor	4817	0.986
87	Janus tyrosine kinase 3 inhibitor	4023	0.985
88	Lck kinase inhibitor	3297	0.963
89	MAP kinase 1 inhibitor	1263	0.968
90	MAP kinase 10 inhibitor	1243	0.980
91	MAP kinase 11 inhibitor	435	0.980
92	MAP kinase 12 inhibitor	139	0.911
93	MAP kinase 14 inhibitor	5624	0.983
94	MAP kinase 3 inhibitor	79	0.880
95	MAP kinase 7 inhibitor	24	0.984
96	MAP kinase 8 inhibitor	1220	0.970
97	MAP kinase 9 inhibitor	966	0.965
98	MAP kinase kinase 1 inhibitor	1079	0.967
99	MAP kinase kinase 4 inhibitor	27	0.902
100	MAP-kinase-activated kinase 2 inhibitor	2014	0.983
101	MAP3K5 inhibitor	89	0.979
102	MAP3K7 inhibitor	76	0.980
103	MAP3K8 inhibitor	907	0.997
104	MDM2 inhibitor	426	0.990
105	MDM4 inhibitor	6	0.982
106	Mcl-1 antagonist	981	0.958
107	Neuronal nitric-oxide synthase inhibitor	711	0.996
108	Neutral endopeptidase inhibitor	1360	0.997
109	Opioid delta receptor agonist	1282	0.993
110	Opioid delta receptor antagonist	2301	0.990
111	P-glycoprotein 1 inhibitor	947	0.943
112	Peroxisome proliferator-activated receptor alpha agonist	2266	0.995
113	Peroxisome proliferator-activated receptor alpha antagonist	289	0.997
114	Phosphatidylinositol 3-kinase alpha inhibitor	6599	0.993
115	Phosphatidylinositol 3-kinase beta inhibitor	1489	0.993
116	Phospholipase C gamma 1 inhibitor	3	0.933
117	Phospholipase D1 inhibitor	95	0.992
118	Phospholipase D2 inhibitor	94	0.997
119	Platelet activating factor beta antagonist	3227	0.976

120	Poly(ADP-ribose) polymerase 1 inhibitor	1559	0.994
121	Polycystin-1 inhibitor	53	0.968
122	Prostaglandin EP4 agonist	167	1.000
123	Prostaglandin EP4 antagonist	266	0.992
124	Protein kinase (CK1) inhibitor	920	0.952
125	Protein kinase (CK2) alpha inhibitor	523	0.958
126	Protein kinase B beta inhibitor	643	0.973
127	Protein kinase B gamma inhibitor	641	0.978
128	Protein kinase C beta inhibitor	703	0.984
129	Protein kinase C delta inhibitor	1194	0.971
130	Protein kinase C delta stimulant	20	1.000
131	Protein kinase C epsilon inhibitor	899	0.991
132	Protein kinase C eta inhibitor	308	0.982
133	Protein kinase C gamma inhibitor	531	0.963
134	Protein kinase C theta inhibitor	2595	0.989
135	Protein kinase C zeta inhibitor	163	0.960
136	Protein phosphatase 2A inhibitor	20	0.999
137	Protein phosphatase 2B inhibitor	10	1.000
138	Protein phosphatase PP1 inhibitor	91	0.946
139	Protein-tyrosine kinase p55(blk) inhibitor	250	0.942
140	Protein-tyrosine phosphatase 1B inhibitor	1899	0.983
141	Protein-tyrosine phosphatase 2C inhibitor	262	0.949
142	Protein-tyrosine phosphatase F inhibitor	39	0.989
143	Protein-tyrosine phosphatase G1 inhibitor	44	0.994
144	Protein-tyrosine phosphatase non-receptor type 22 inhibitor	123	0.980
145	Proto-oncogene tyrosine-protein kinase CSK inhibitor	99	0.940
146	Proto-oncogene tyrosine-protein kinase Fyn inhibitor	724	0.923
147	Proto-oncogene tyrosine-protein kinase Kit inhibitor	2477	0.979
148	Proto-oncogene tyrosine-protein kinase Yes inhibitor	127	0.938
149	Pyruvate dehydrogenase kinase inhibitor	1684	0.982
150	RET inhibitor	768	0.955
151	Raf kinase B inhibitor	3319	0.991
152	Raf kinase inhibitor	4227	0.986
153	Retinoic acid alpha receptor agonist	69	0.999
154	Retinoic acid alpha receptor antagonist	254	0.999
155	Retinoid X alpha receptor agonist	145	0.994
156	Retinoid X alpha receptor antagonist	267	0.997
157	Ribosomal protein S6 kinase 2 inhibitor	13	0.945
158	Ribosomal protein S6 kinase alpha 1 inhibitor	131	0.945
159	Ribosomal protein S6 kinase alpha 3 inhibitor	566	0.943
160	Ribosomal protein S6 kinase alpha 5 inhibitor	494	0.970

161	SMAD3 inhibitor	20	0.890
162	Serine/threonine-protein kinase WEE1 inhibitor	320	0.996
163	Serum-glucocorticoid regulated kinase 1 inhibitor	78	0.924
164	Smooth muscle myosin light chain kinase inhibitor	125	0.936
165	T-cell protein-tyrosine phosphatase inhibitor	336	0.991
166	TIE-2 agonist	6	1.000
167	TIE-2 antagonist	1807	0.979
168	TRKA antagonist	780	0.956
169	Telomerase inhibitor	640	0.974
170	Telomerase stimulant	6	1.000
171	Topoisomerase II alpha inhibitor	69	0.965
172	Transcription factor AP-1 inhibitor	300	0.974
173	Transcription factor NF kappa B1 inhibitor	25	0.993
174	Transcription factor RelA inhibitor	45	0.979
175	Transcription factor STAT3 inhibitor	290	0.919
176	Transcription factor STAT6 inhibitor	72	0.995
177	Transforming growth factor beta 1 agonist	14	0.938
178	Transforming growth factor beta 1 antagonist	935	0.994
179	Transitional endoplasmic reticulum ATPase inhibitor	105	0.991
180	Tyrosine-protein kinase ABL2 inhibitor	13	0.948
181	Tyrosine-protein kinase receptor FLT3 inhibitor	2721	0.970
182	Vascular endothelial growth factor 2 antagonist	11846	0.970
183	Vitamin D receptor agonist	102	1.000
184	Vitamin D receptor antagonist	131	0.987
185	c-Src kinase inhibitor	3699	0.971
186	cAMP-dependent protein kinase alpha catalytic subunit inhibitor	438	0.958
